# Synthesis of a one-handed helical polythiophene: a new approach using an axially chiral bithiophene with a fixed *syn*-conformation†
†Electronic supplementary information (ESI) available: Detailed experimental procedures, characterizations of molecules/polymers and additional spectroscopic, chromatographic and computational data. See DOI: 10.1039/c9sc00342h


**DOI:** 10.1039/c9sc00342h

**Published:** 2019-04-03

**Authors:** Tomoyuki Ikai, Kokoro Takayama, Yuya Wada, Serena Minami, Chanokporn Apiboon, Ken-ichi Shinohara

**Affiliations:** a Graduate School of Natural Science and Technology , Kanazawa University , Kakuma-machi , Kanazawa 920-1192 , Japan . Email: ikai@se.kanazawa-u.ac.jp; b School of Materials Science , Japan Advanced Institute of Science and Technology (JAIST) , 1-1 Asahi-dai , Nomi 923-1292 , Japan

## Abstract

We report an optically active polythiophene capable of forming a one-handed helically folded conformation without needing aggregate formation, poor solvent conditions, hydrogen-bonded ion-pair formation or guest addition. The target polythiophene (poly-T_R_) with a static axial chirality in the main chain was synthesized *via* Stille coupling copolymerization of a glucose-linked chiral 5,5′-dibromobithiophene with 2,5-bis(stannyl)thiophene. Poly-T_R_ showed a characteristic circular dichroism and circularly polarized luminescence, which were completely different to those observed for an analogous polymer (poly-Ph_R_) and the corresponding unimer/dimer model compounds. This chiroptical study, combined with the results of all-atom molecular dynamics simulations, revealed that poly-T_R_ can fold into a left-handed helical conformation under good solvent conditions. Partial conformational regulation derived from the fixed *syn*-conformation of the chiral bithiophene unit was considered a key factor in producing the one-handed helical polythiophene.

## Abstract 2

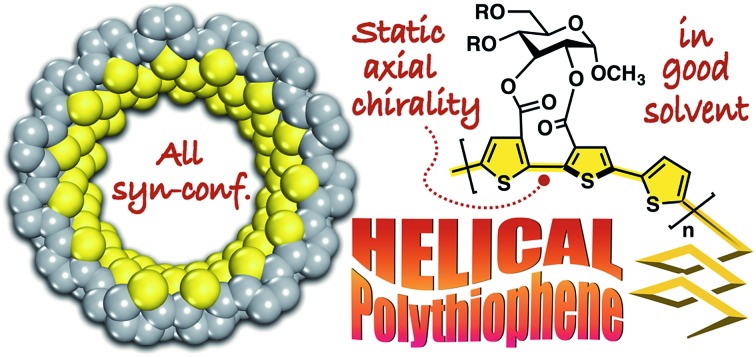
An optically active polythiophene, which can fold into a one-handed helical conformation under good solvent conditions, has been developed.

## Introduction

A simple poly(thiophene-2,5-diyl) without any pendant groups was successfully synthesized *via* chemical polymerization in 1980.^1^,^2^ In the last four decades, much research effort has been focused on developing a range of functional polythiophenes (herein referring to poly(thiophene-2,5-diyl)s, unless otherwise noted) using various polymerization techniques and/or postpolymerization modifications.^3^–^9^ Polythiophenes are now recognized as an important class of π-conjugated polymers and have been extensively studied for applications in organic devices and functional materials, including as photovoltaics, field-effect transistors, light emitting diodes, magneto-optics, actuators and chemical/biological sensors.^10^–^15^ This practical utility arises from their excellent semiconducting, optical and magnetic features, in addition to substantial environmental stability and mechanical strength.

Chiral polythiophenes containing optically active components somewhere in the molecular structures have also attracted considerable attention in the fields of supramolecular chemistry, because these polymers can easily self-assemble into helical aggregates through intermolecular π–π interactions under poor solvent conditions or in a properly treated film.^16^–^22^ These supramolecular chiral aggregates often show more intense chiroptical properties (that is, optical rotation, circular dichroism (CD) and circularly polarized luminescence (CPL)) than those in a molecularly dispersed state. On the other hand, Cui *et al.* have predicted the possible existence of a helically folded polythiophene through semi-empirical calculations.^23^ In fact, several groups have succeeded in preparing chiral polythiophenes with macromolecular helicity rather than supramolecular helicity. Wennerström and Inganäs *et al.* reported that polythiophenes bearing ionic pendant groups could form a one-handed helical conformation in aqueous solutions, where hydrogen-bonded ion-pair formations within the polymer chain played important roles in helix formation.^24^–^27^ Shinkai *et al.* and Inganäs *et al.* found that a helical conformation was induced in ionic polythiophenes upon complexation with guest molecules and polymers.^28^–^35^ Meijer *et al.* and Koeckelberghs *et al.* also reported nonionic helical polythiophenes that could fold only under poor solvent conditions containing water or methanol, probably due to hydrophobic interactions.^36^,^37^ Furthermore, *ortho*-linked polythiophenes, poly(thiophene-2,3-diyl)s, can adopt a helical conformation, as evidenced by scanning tunneling microscopy imaging, although helicity control was not achieved owing to an achiral molecular design.^38^ In each case, specific environments or situations have been required to provide polythiophene backbones with macromolecular helicity, while helix formation has not been achieved in a molecularly dispersed state under good solvent conditions. The environment-independent helix-forming feature of polythiophenes is advantageous from a practical perspective, because careful consideration of extrinsic influences, such as concentration, temperature and solvent, is not required, which simplifies device manufacture and expands the application possibilities.

**Fig. 1:**
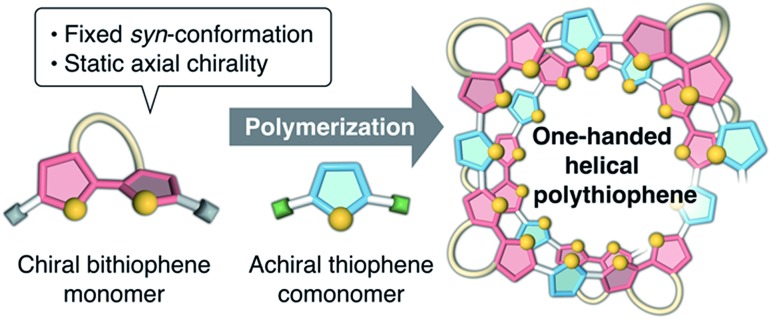
Schematic illustration of the synthetic approach to one-handed helical polythiophene using the fixed *syn*-conformation of a chiral bithiophene with static axial chirality.

Recently, we synthesized a series of optically active π-conjugated polymers, poly(arylene ethynylene)s, containing a d-glucose-linked biaryl unit as a key structural element in the main chain.^39^–^43^ We demonstrated that some of these polymers possessing favorable combinations of backbone and pendant structures folded into helical conformations with a preferred-handedness. In this work, we report a new synthetic approach to one-handed helical polythiophene using the fixed *syn*-conformation of a d-glucose-linked bithiophene unit with static axial chirality (Fig. 1).^41^ The one-handed helix formation of the resulting polythiophene in a molecularly dispersed state, particularly under good solvent conditions, was investigated using both chiroptical analysis under various conditions and an all-atom molecular dynamics (MD) study.

## Results and discussion

### Synthesis

**Fig. 2:**
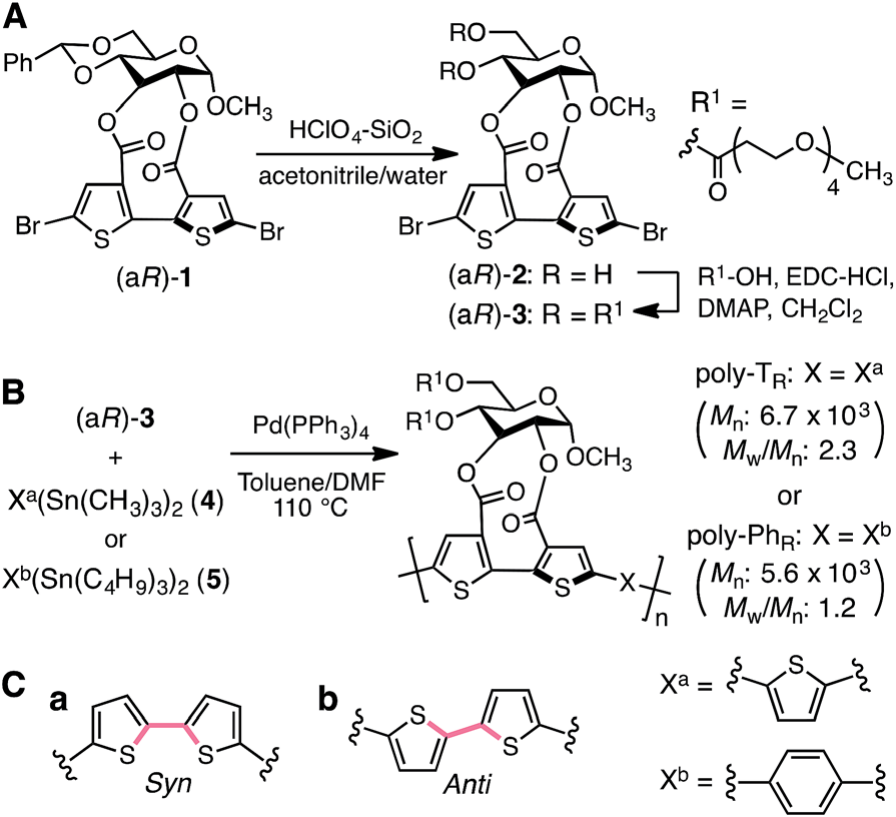
Synthesis of (A) optically active bithiophene monomer (a*R*)-**3** and (B) π-conjugated polymers poly-T_R_ and poly-Ph_R_. (C) *Syn*- (a) and *anti*- (b) conformations of a bithiophene unit.

**Chart 1:**
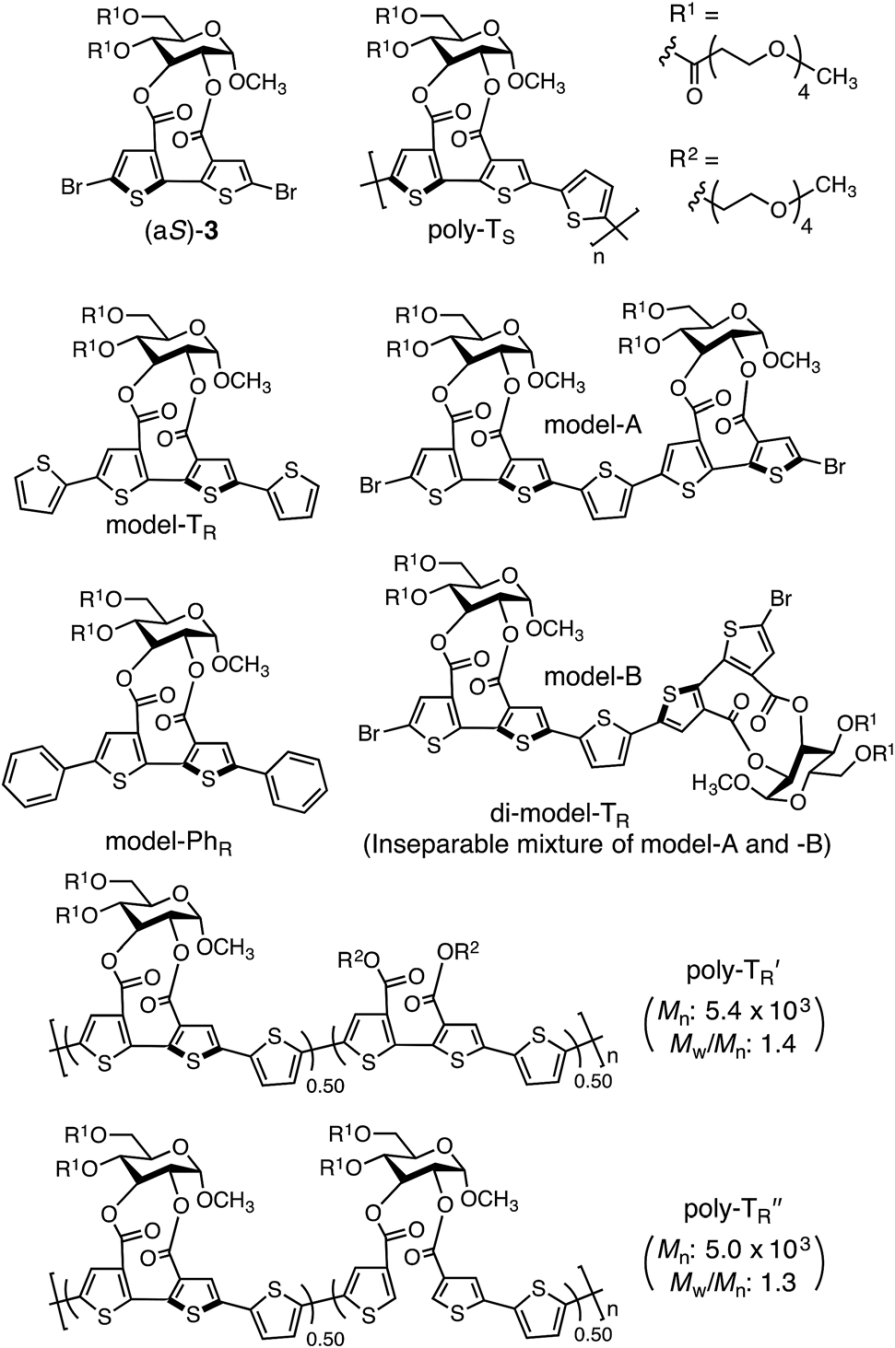


The synthetic routes used to prepare optically active bithiophene monomer (a*R*)-**3** and π-conjugated polymers poly-T_R_ and poly-Ph_R_ are summarized in Fig. 2. Monomer (a*R*)-**3**, containing point and axial chirality derived from glucose and biaryl units, respectively, was synthesized from a previously reported 5,5′-dibromobithiophene derivative, (a*R*)-**1**,^41^ through acetal deprotection followed by esterification at the 4- and 6-positions of the glucose unit. The obtained (a*R*)-**3** was copolymerized with **4** or **5***via* Stille coupling in toluene/*N*,*N*-dimethylformamide (4 : 1, v/v) at 110 °C. The optically active polymers, poly-T_R_ and poly-Ph_R_, containing 2,5-thienylene and *p*-phenylene comonomer units, respectively, were obtained in moderate yields (>57%), with molecular masses (*M*_n_) greater than 5.6 × 10^3^ g mol^–1^, as estimated by size-exclusion chromatography (SEC). The copolymerization of **4** and (a*S*)-**3**, a diastereomer of (a*R*)-**3** with opposite axial chirality (see Chart 1), was also performed using the same method. However, as the resulting poly-T_S_ was almost insoluble in common organic solvents, including chloroform and tetrahydrofuran, it could not be further characterized. Monomeric model compounds model-T_R_ and model-Ph_R_ (Chart 1), related to poly-T_R_ and poly-Ph_R_, respectively, were synthesized for comparative studies (Scheme S1†).

### Chiroptical properties

**Fig. 3:**
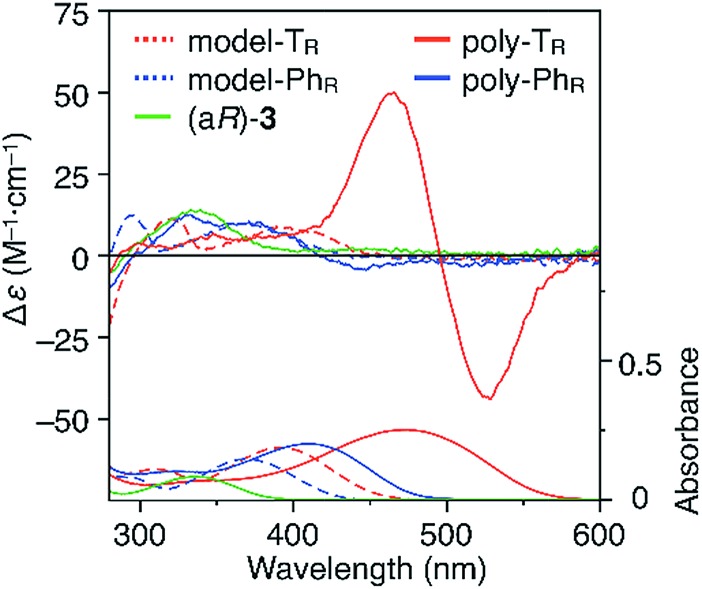
CD and absorption spectra of (a*R*)-**3**, model-T_R_, model-Ph_R_, poly-T_R_ and poly-Ph_R_ in chloroform at 25 °C. [Glucose unit] = 1.0 × 10^–4^ M.

CD and absorption spectra of the monomer, unimer models and polymers in chloroform are shown in Fig. 3. Poly-T_R_ showed a clear bisignated Cotton effect in the absorption region of the polymer backbone (400–580 nm) and its CD intensity was much larger than those observed for (a*R*)-**3** and the corresponding model-T_R_. Although the absorption regions were different due to differing π-conjugation lengths and a simple comparison was difficult, this CD comparison indicated that the characteristic Cotton effect observed for poly-T_R_ did not arise from only the chirality of the d-glucose-based monomeric unit. Considering the chiroptical investigation of previously reported chiral polythiophenes,^16^ the poly-T_R_'s CD was presumed to derive from either supramolecular chirality induced in the polymer aggregate or conformational chirality induced in a single polymer chain with a regular higher-order structure. To gain further insight into the CD origin, we performed chiroptical measurements of poly-T_R_ under several conditions. When the poly-T_R_ concentration in chloroform was varied in the range of 0.01–1.0 mM, almost no concentration dependence was observed in the (chir)optical properties (Fig. S1†). Furthermore, the absorbance and CD intensities in chloroform did not change after passing through a membrane filter with a pore size of 0.20 μm (Fig. S2†). These results suggested that the intense CD signal of poly-T_R_ was derived from a specific secondary structural formation within a single polymer chain, rather than the formation of polymer aggregates through intermolecular interactions. Notably, the bisignated CD spectral pattern of poly-T_R_ closely resembled those of previously reported polythiophenes with one-handed helical conformations,^24^–^27^ in which all torsion angles between thiophene rings were regulated to a *syn*-conformation, as shown in Fig. 2C(a). Therefore, the π-conjugated backbone of poly-T_R_ was considered to fold into a helical conformation. Owing to the relationship between the Cotton-effect signs of the reported helical polythiophenes and their helical senses,^24^–^27^ the poly-T_R_ backbone, which showed a negative first Cotton effect, most likely had left-handed helicity. This was also supported by a molecular modeling study, as discussed in a later section.

**Fig. 4:**
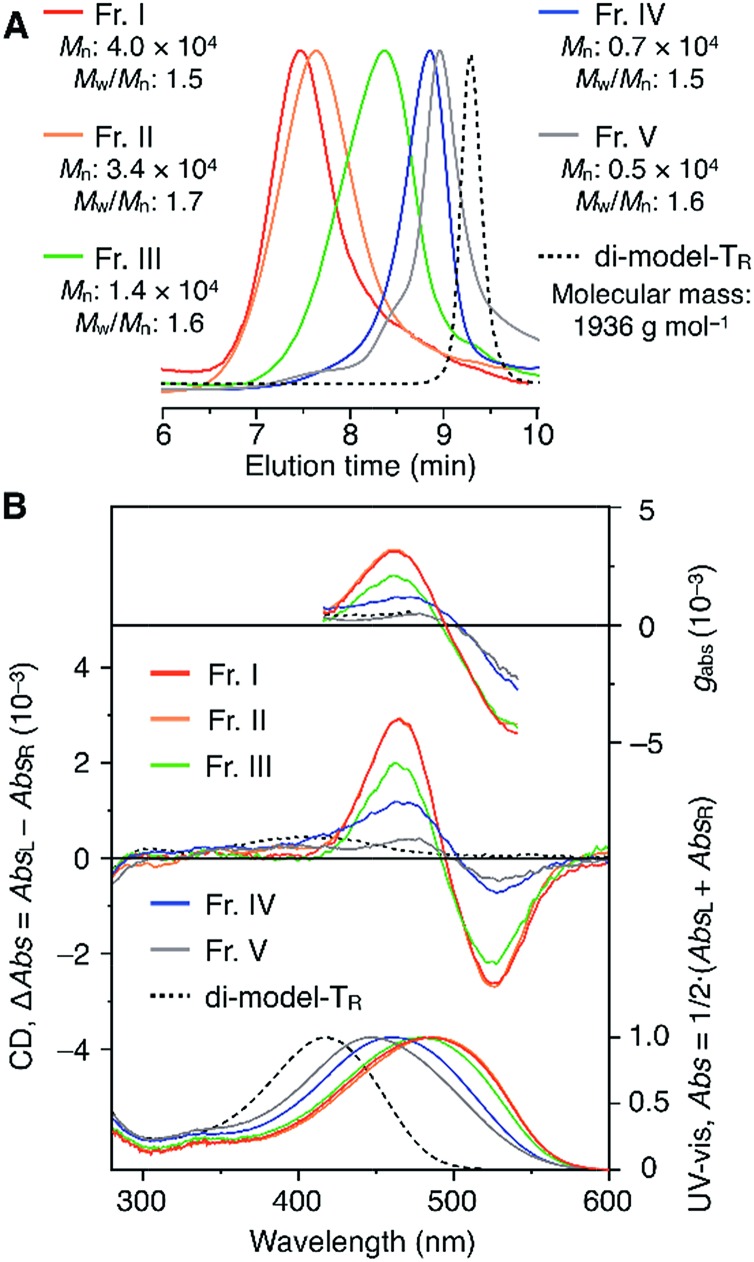
(A) SEC traces of fractionated components of poly-T_R_ with different molecular masses (eluent, chloroform; polystyrene standards). (B) Molecular mass dependence of the CD and absorption spectra of poly-T_R_ in chloroform at 25 °C.

The molecular mass dependence of the CD intensity was investigated using poly-T_R_ with different molecular masses (*M*_n_) in the range of 0.5 × 10^4^ to 4.0 × 10^4^ g mol^–1^ (Fig. 4), prepared by SEC fractionation (Fig. S3†). The results of the dimer model (di-model-T_R_, see Chart 1), which contained a regioisomeric mixture of model-A and model-B, are also shown. The CD intensity of poly-T_R_ clearly increased with increasing molecular mass and reached an almost constant value when *M*_n_ was above 3.4 × 10^4^ g mol^–1^ (corresponding to approximately 37 repeating units) (Fig. 4B). This molecular mass dependence of the chiroptical properties is characteristic of optically active polymers with one-handed macromolecular helicity,^21^,^44^ because the corresponding oligomers cannot gain sufficient stabilization energy to maintain the helically folded state. Therefore, this CD study provided further evidence that the poly-T_R_ backbone possessed a specific secondary structure in solution. We also confirmed that the absorption dissymmetry factor (*g*_abs_ = Δ*ε*/*ε*) of poly-T_R_ was mostly retained in the temperature range of –10 to 50 °C (Fig. S4†), indicating that the helical conformation of poly-T_R_ was thermally stable to some extent. Moreover, intense CD signals derived from the helical chirality of poly-T_R_ were observed in various solvent systems, including tetrahydrofuran and chlorobenzene, which are typically good solvents for polythiophenes (Fig. S5†). To our knowledge, this is the first example of a molecularly dispersed one-handed helical polythiophene existing under good solvent conditions, whose backbone conformation was mostly maintained regardless of the exterior environmental conditions, such as concentration, temperature and solvent. In sharp contrast, poly-Ph_R_ showed a relatively weak CD signal with an intensity and pattern comparable to those of the corresponding model-Ph_R_ (Fig. 3). Furthermore, the chiroptical property of poly-Ph_R_ was almost independent on the molecular mass (Fig. S6†), suggesting that the poly-Ph_R_ backbone did not form a specific higher-order structure. As we have previously reported for an analogous glucose-linked poly(arylene ethynylene),^45^ the phenylene units might not provide enough cooperative π–π interactions within the main chains, which are necessary to stabilize the helix structure. Poly-T_R_ analogues, 
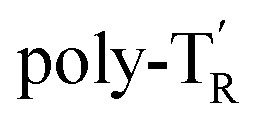
 and 
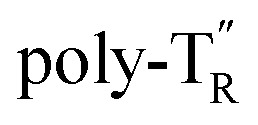
 (see Chart 1), which contained an achiral bithiophene unit capable of free internal rotation and a d-glucose-appended unit without a covalent bond between thiophene rings, respectively, did not show apparent CD signals (Fig. S7†). This result clearly means that a periodic arrangement of the axially chiral bithiophene units with a fixed *syn*-conformation presented here is important for helical folding.

**Fig. 5:**
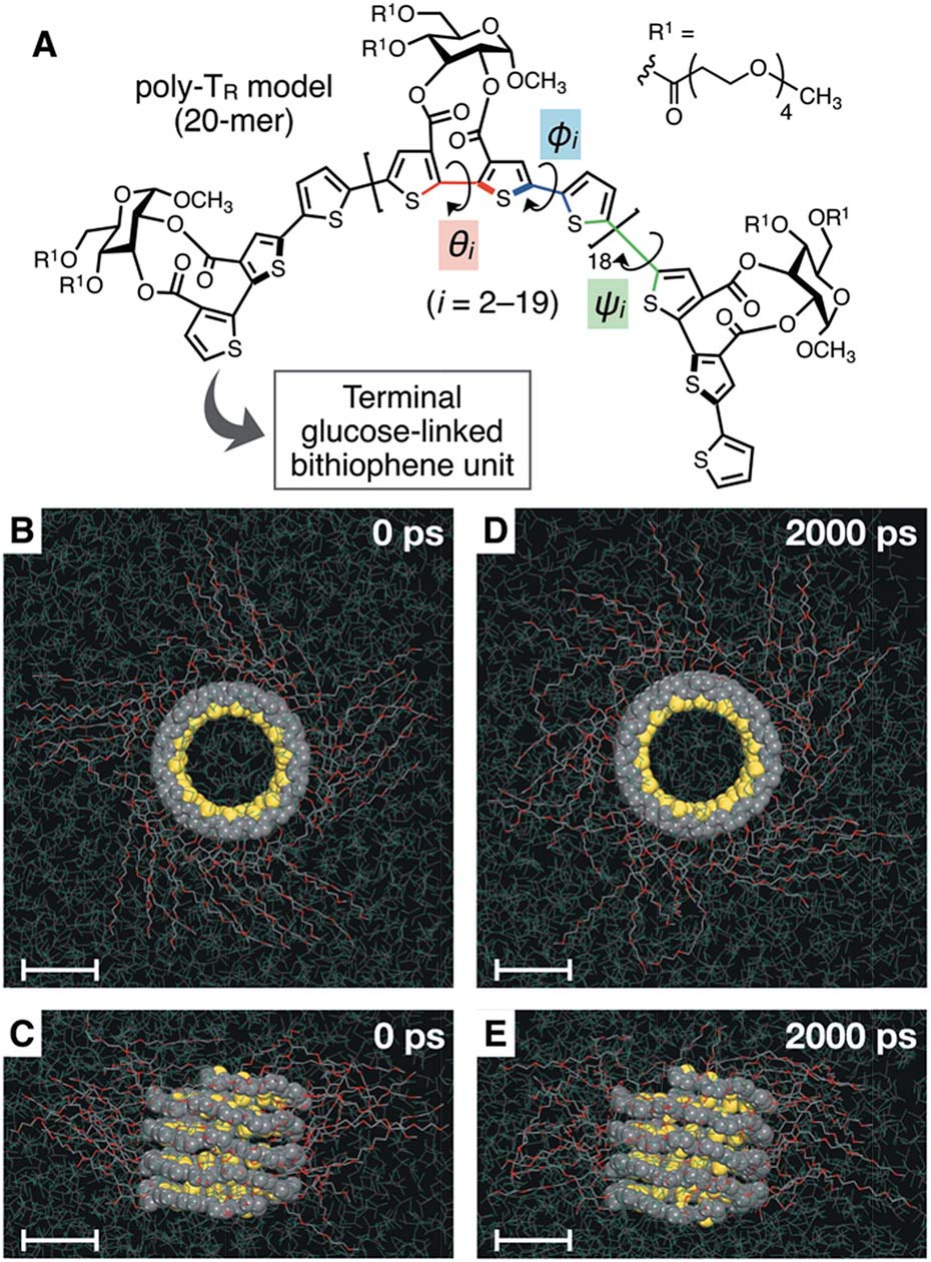
(A) Structure of the poly-T_R_ model (20-mer) used for the computational study. (B, D) Top view and (C, E) side view of the molecular model of left-handed helically folded poly-T_R_ in chloroform at (B, C) 0 ps and (D, E) 2000 ps in an all-atom MD simulation after equilibration at 298 K, represented by space-filling (polythiophene backbone) and stick (side chain) models. The chloroform solvent molecules are represented by line models and their hydrogen atoms are omitted to simplify the view. All scale bars represent 1 nm.

**Fig. 6:**
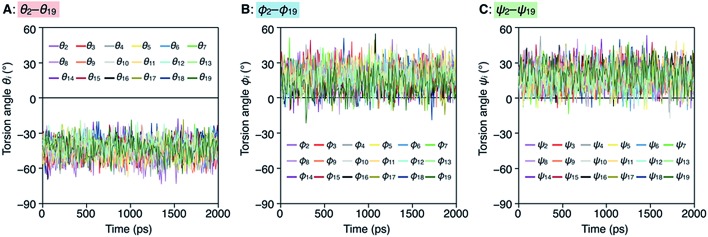
Plots of *θ*_*i*_ (A), *φ*_*i*_ (B) and *ψ*_*i*_ (C) as a function of calculation time.

To better understand the helix conformation of poly-T_R_, its all-atom MD simulation in chloroform was conducted using the corresponding model with a degree of polymerization of 20 (Fig. 5A). The initial structure with a left-handed helical backbone was constructed such that all neighboring thiophene pairs were arranged in *syn*-conformations. After equilibration at 298 K (see ESI†), the simulation in the microcanonical (NVE) ensemble was conducted for 2000 ps as the production run (Video S1 and S2†). The molecular models in the initial (0 ps) and final (2000 ps) states are shown in Fig. 5B–E. The molecular model in the middle (1000 ps) stage is shown in Fig. S8.† This simulation clearly demonstrated that the left-handed helically folded conformation, in which a single turn of the helix was composed of approximately 15 thiophene rings (Fig. S9†) and all sulfur atoms were arranged inside the helical cavity, remained unchanged after 2000 ps and was probably the favorable conformation for poly-T_R_ in chloroform. This computer-generated helix model was similar to that proposed by Kiriy *et al.*^46^ Furthermore, the time-dependent changes of three torsion angles (*θ*_*i*_, *φ*_*i*_ and *ψ*_*i*_, see Fig. 5A) between the two thiophene planes in the *i*th repeating units from the terminal glucose-linked bithiophene unit were plotted, as shown in Fig. 6A–C. To reduce the influence of the chain ends, the results of the 18 repeating units (*i* = 2–19) were used for the discussion. The average torsion angles (*θ̄*_*i*_, *ϕ̄*_*i*_ and *ψ̄*_*i*_) in the 20-mer model and their standard deviations (SD) are summarized in Table S1–S3.† This statistics data revealed that the torsion angles in the glucose-linked bithiophene units (*θ*_*i*_) were always negative, while the other two (*φ*_*i*_ and *ψ*_*i*_) were mostly positive with a few exceptions. In other words, three axial chirality arising in the repeating unit was not identical, and one (a*R*)- and two (a*S*)-configurations were arranged in the main chain, essentially in an alternating manner. Although the axial chirality in the main chain was inconsistent, all *θ*_*i*_, *φ*_*i*_ and *ψ*_*i*_ values fell within the range of –90° to +90°, suggesting that all pairs of neighboring thiophene planes perfectly retained their *syn*-conformations throughout the calculation period. For comparison, the right-handed helically folded model of poly-T_R_ with all-*syn* conformations was constructed as an initial structure and its all-atom MD simulation was conducted under the same conditions (Video S3 and S4†). In sharp contrast to the left-handed helix model, several *anti*-conformations were produced in the right-handed polythiophene backbones and the regular higher-order structure was not maintained during the calculation (Fig. S10†). This simulation supported that the plausible helix sense of poly-T_R_ was left-handed, as described above.

### Circularly polarized luminescence properties

**Fig. 7:**
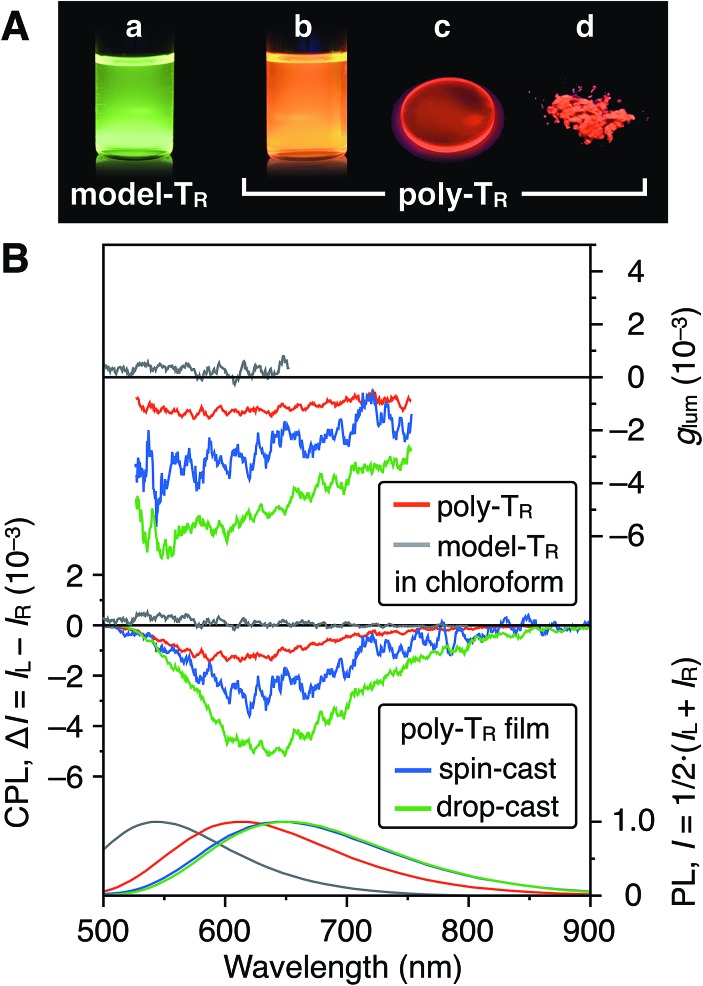
(A) Photograph of chloroform solutions of (a) model-T_R_ and (b) poly-T_R_, and (c) the drop-cast film and (d) powder of poly-T_R_ under irradiation at 365 nm. (B) PL (bottom), CPL (middle) and *g*_lum_ (top) spectra of poly-T_R_ and model-T_R_ in chloroform ([Glucose unit] = 1.0 × 10^–4^ M) and the poly-T_R_ films at room temperature. Excitation wavelength (*λ*_ex_), 365 nm.

Fig. 7A shows photographs of poly-T_R_ in chloroform and the solid state under 365 nm irradiation. Poly-T_R_ exhibited an apparent orange photoluminescence (PL) in both states, which was attributed to the polythiophene backbone. The fluorescence quantum yield of poly-T_R_ in chloroform was determined to be 5%. The above chiroptical and PL properties of the optically active poly-T_R_ allowed us to investigate its CPL performance. The PL, CPL and luminescence dissymmetry factor (*g*_lum_) spectra of poly-T_R_ in chloroform are shown in Fig. 7B. Here, *g*_lum_ = 2(*I*_L_ – *I*_R_)/(*I*_L_ + *I*_R_), where *I*_L_ and *I*_R_ are the PL intensities of the left- and right-handed circularly polarized light, respectively. Poly-T_R_ in chloroform emitted right-handed circularly polarized light in the fluorescence region of the π-conjugated backbone, with the maximum |*g*_lum_| value estimated to be 1.6 × 10^–3^, which was the same order as the maximum |*g*_abs_| value of poly-T_R_ in chloroform (4.3 × 10^–3^). This means that the helical structure in the ground state was likely retained in the excited state. Furthermore, we confirmed that the |*g*_lum_| of poly-T_R_ was close to that observed for a previously reported helical polythiophene (4.5 × 10^–3^) prepared through complexation with a polysaccharide.^33^ As expected from the CD comparison discussed above, the CPL performance of poly-T_R_ was superior to that of model-T_R_. Thus, we concluded that the helical conformation of the polythiophene backbone was of key importance for producing a moderate CPL. This statement was also supported by the fact that poly-Ph_R_ did not produce an apparent CPL signal (Fig. S11†). Next, to investigate the solid-state CPL performance, we prepared poly-T_R_ films by drop- and spin-coating the chloroform solution onto quartz plates.^47^ The emission maximum wavelength in the film state was red-shifted by approximately 50 nm compared with the result in chloroform, which was due to elongation of the π-conjugation length. Furthermore, the poly-T_R_ films showed better CPL performances than poly-T_R_ in chloroform, with the maximum |*g*_lum_| (6.9 × 10^–3^) achieved by the drop-cast film in this study. The same tendency was also observed in the CD comparison between the solution and solid states (Fig. S14–S16†). A certain regular arrangement of helically folded poly-T_R_ and its intermolecular couplet might be related to higher *g*_abs_ and *g*_lum_ values being achieved in the film state than in chloroform.

## Conclusions

We have synthesized a new optically active polythiophene derivative (poly-T_R_) containing d-glucose-linked axially chiral bithiophene units with a *syn*-conformation in the main chain. Based on the comprehensive chiroptical study using poly-T_R_, its analogous polymer and model compounds, we concluded that the poly-T_R_ backbone possessed a specific secondary structure (helix) even under good solvent conditions, such as in chloroform, THF and chlorobenzene. Furthermore, all-atom MD simulations revealed that the probable helix sense of poly-T_R_ was left-handed and a single helical turn was composed of approximately 15 thiophene rings. We also demonstrated that poly-T_R_ can emit right-handed circularly polarized light due to the macromolecular helical chirality, with a maximum |*g*_lum_| value of 6.9 × 10^–3^ reached in the film state. We believe that this helical polythiophene with an environment-independent folding feature represents a new category of advanced functional materials and is attractive for applications to organic electronics, sensors and host–guest systems through further modification of the backbone and pendant units. Further studies towards these goals are underway in our laboratory.

## Conflicts of interest

There are no conflicts to declare.

## Supplementary Material

Supplementary information

Supplementary movie

Supplementary movie

Supplementary movie

Supplementary movie
