# Supplementary material for: Synthesis of a one-handed helical polythiophene: a new approach using an axially chiral bithiophene with a fixed syn-conformation
Source: Chem Sci. 2019 Apr 3;10(18):4890–5. doi: 10.1039/c9sc00342h (PMC6520921; doi:10.1039/c9sc00342h)
Supplement: Supplementary file 1 [file SC-010-C9SC00342H-s001.pdf]

Electronic Supplementary Material (ESI) for Chemical Science.
This journal is © The Royal Society of Chemistry 2019

(Supplementary Information)

# Synthesis of a one-handed helical polythiophene: a new approach using an axially chiral bithiophene with a fixed *syn*-conformation

Tomoyuki Ikai,[a,*] Kokoro Takayama,[a] Yuya Wada,[a] Serena Minami,[a] Chanokporn Apiboon[a] and Ken-ichi Shinohara[b]

[a]*Graduate School of Natural Science and Technology, Kanazawa University, Kakuma-machi, Kanazawa 920-1192, Japan*

[b]*School of Materials Science, Japan Advanced Institute of Science and Technology (JAIST), 1-1 Asahi-dai, Nomi 923-1292, Japan*

*To whom correspondence should be addressed. E-mail: ikai@se.kanazawa-u.ac.jp.

**Table of content:**

**1. Materials** ······ S-2

**2. Instruments** ······ S-2

**3. Synthesis** ······ S-3

**4. All-atom molecular dynamics simulation** ······ S-9

**Supporting data** ······ S-10

**NMR spectral data** ······ S-24

**Captions for supporting videos** ······ S-35

**References** ······ S-35

## 1. Materials

Anhydrous solvents (acetonitrile, dichloromethane, toluene and *N*,*N*-dimethylformamide (DMF)) and common organic solvents were purchased from Kanto Kagaku (Tokyo, Japan). 2,5-Bis(trimethylstannyl)thiophene (**4**), 2-(tributylstannyl)thiophene (**4b**), 1,4-bis(tributylstannyl)benzene (**5**), trimethyl(phenyl)tin (**5b**) and *N*,*N*-dimethyl-4-aminopyridine (DMAP) were from Sigma-Aldrich (St. Louis, MO, USA). 4,7,10,13-Tetraoxatetradecanoic acid was from Broadpharm (San Diego, CA, USA). 1-Ethyl-3-(3-dimethylaminopropyl)carbodiimide hydrochloride (EDC-HCl) was purchased from Wako Pure Chemical Industries (Osaka, Japan). Tetrakis(triphenylphosphine)palladium(0) ($Pd(PPh_3)_4$) was purchased from Nacalai (Kyoto, Japan). Tetraethylene glycol monomethyl ether was from Tokyo Kasei Kogyo (TCI) (Tokyo, Japan). Silica supported perchloric acid ($HClO_4$–$SiO_2$)[S1], 5,5′-dibromo-2,2′-bithiophene-3,3′-dicarboxylic acid[S2] and **6b**-prec-1[S2] were prepared according to literature procedures.

## 2. Instruments

NMR spectra were taken on a JNM-ECA 500 (JEOL, Tokyo, Japan) (500 MHz for $^{1}H$, 126 MHz for $^{13}C$) or a JNM-ECA 600 (JEOL) (151 MHz for $^{13}C$) spectrometer in $CDCl_3$ using tetramethylsilane as the internal standard. Recycling preparative HPLC was performed with an LC-9201 liquid chromatography (JAI, Tokyo, Japan) equipped with a UV-2075 UV–visible detector (JASCO, Tokyo, Japan) at room temperature. HPLC columns, JAIGEL-1H and JAIGEL-2H, were connected in series, where chloroform was used as the eluent. IR spectra were obtained using a JASCO IR-4700 Fourier Transform spectrophotometer with a KBr pellet. The molecular weights ($M_w$) and distributions ($M_w/M_n$) of the polymers were estimated using size-exclusion chromatography (SEC) equipped with a TSKgel Multipore$H_{XL}$-M column (Tosoh, Tokyo, Japan), a JASCO PU-2080 Intelligent HPLC pump and a JASCO UV-970 UV/VIS detector at 254 nm, where chloroform was used as the eluent. The molecular weight calibration curve was obtained with polystyrene standards (Tosoh). High-resolution mass spectra (HRMS) were recorded on a JEOL JMS-700 spectrometer with fast atom bombardment (FAB) as the ionization technique. Absorption and circular dichroism (CD) spectra were measured using a JASCO V-570 (a scanning rate of 200 nm $min^{-1}$ and a bandwidth of 1.0 nm) and a JASCO J-720L (a scanning rate of 100 nm $min^{-1}$ and a bandwidth of 1.0 nm) spectrometers, respectively, with a quartz cell of 0.10, 1.0 or 10 mm path length (UV-grade) (GL Sciences, Tokyo, Japan). The temperature was controlled using a JASCO ETC-505T (absorption spectroscopy) and a JASCO PTC-348WI apparatus (CD spectroscopy). The optical rotation was measured at 25 °C with a JASCO P-1030 polarimeter. The fluorescence quantum yields excited at 365 nm were evaluated using a JASCO FP-6600 spectrometer attached with a JASCO ILF-533 integrating sphere (diameter 10 cm).

Photoluminescence (PL) and circularly polarized luminescence (CPL) spectra in solution states were recorded at room temperature on a JASCO CPL-300 with 1.0 mm in path length quartz cell (GL Sciences, UV-grade). A drop- and spin-coated films prepared on a quartz substrate (Daico MFG, USQ-grade) from a chloroform solution of polymers (*ca.* 10 mg mL$^{-1}$) was used for solid-state spectral measurements. A scanning rate of 100 nm min$^{-1}$, an excitation slit width of 3000 μm, a monitoring slit width of 3000 μm, a response time of 4 seconds and 2 times (solution state) or single (film state) accumulation were employed.

## 3. Synthesis

Model compounds (model-T$_R$, model-Ph$_R$ and di-model-T$_R$), (a*S*)-**3** and ternary copolymers (poly-T$_R$′ and poly-T$_R$″) were synthesized according to Scheme S1.

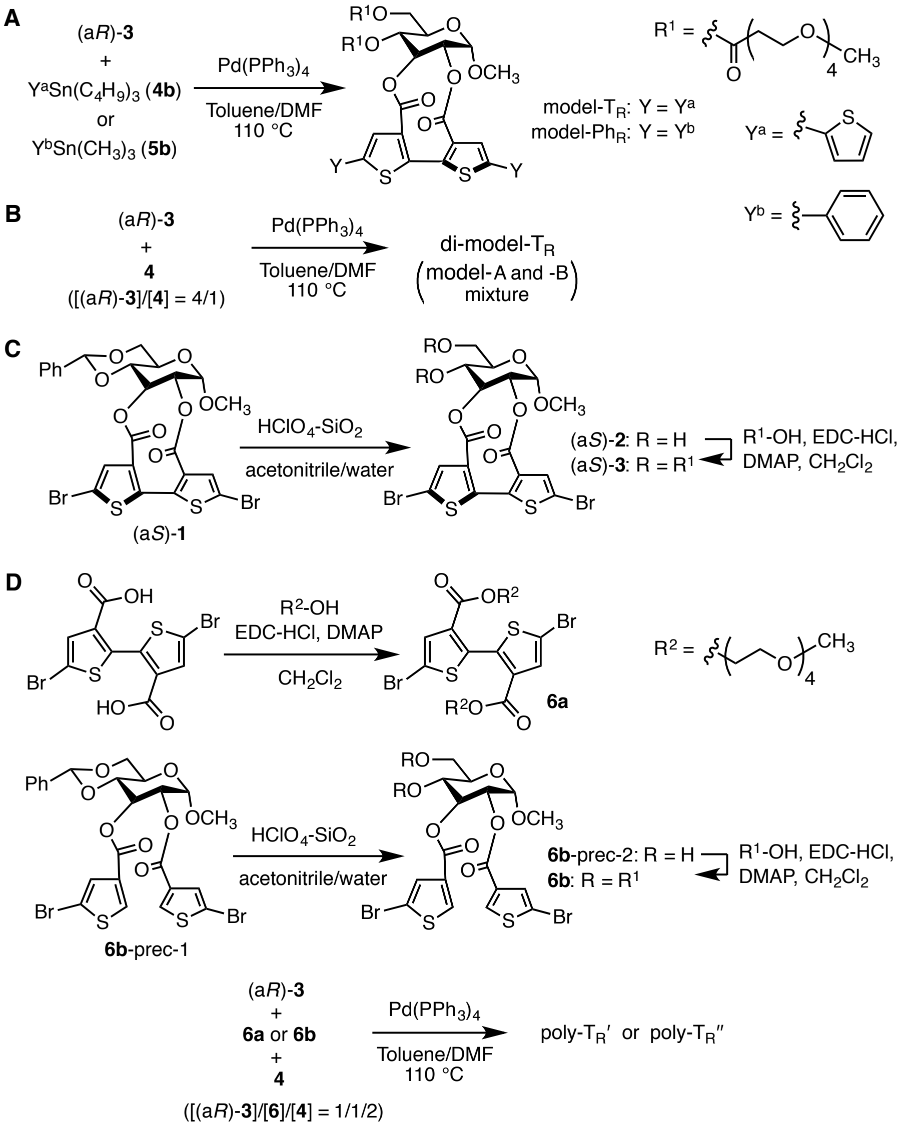


**Scheme S1** Synthesis of (A) the unimer model compounds (model-T$_R$ and model-Ph$_R$), (B) the dimer model compound (di-model-T$_R$), (C) (a*S*)-**3** and (D) the ternary copolymers (poly-T$_R$′ and poly-T$_R$″).

(a*R*)-**1** and (a*S*)-**1**. A diastereomeric mixture containing (a*R*)-**1** and (a*S*)-**1** was prepared according to a literature procedure[S2] and obtained in 67% yield as a pale yellow solid. The obtained diastereomers were resolved by recycling preparative HPLC on JAIGEL-2HR (column dimensions: 60 cm × 2.0 cm (i.d.); eluent: chloroform; flow rate 9 mL $min^{-1}$; temperature *ca.* 20 °C) to give (a*R*)-**1** and (a*S*)-**1** as a pale yellow solid. (a*R*)-**1**: $^1$H NMR (500 MHz, $CDCl_3$, rt): $\delta$ 7.50 (br, 2H, ArH), 7.39 (br, 4H, ArH), 7.06 (s, 1H, ArH), 5.57 (s, 1H, CH), 4.94 (br, 3H, CH), 4.34 (br, 1H, CH), 3.82-3.90 (m, 3H, CH, $CH_2$), 3.36 (s, 3H, $CH_3$). (a*S*)-**1**: $^1$H NMR (500 MHz, $CDCl_3$, rt): $\delta$ 7.63 (s, 1H, ArH), 7.57 (s, 1H, ArH), 7.32-7.37 (m, 5H, ArH), 5.34 (s, 1H, CH), 5.06 (t, *J* = 10.0 Hz, 1H, CH), 4.79 (d, *J* = 3.4 Hz, 1H, CH), 4.35 (dd, *J* = 10.0, 3.7 Hz, 1H, $CH_2$), 4.25 (dd, *J* = 10.0, 4.5 Hz, 1H, $CH_2$), 3.75-3.80 (m, 1H, CH), 3.64 (t, *J* = 10.3 Hz, 1H, CH), 3.32 (s, 3H, $CH_3$), 2.88 (t, *J* = 9.5 Hz, 1H, CH).

(a*R*)-**2**. To a solution of (a*R*)-**1** (1.62 g, 2.46 mmol) in acetonitrile/water (300:1, v/v) (66 mL) was added $HClO_4$–$SiO_2$ (2.2 g). After stirring at room temperature for 1 h, the reaction mixture was diluted with ethyl acetate and passed through a short pad of silica gel using ethyl acetate as the eluent to remove $HClO_4$–$SiO_2$. After removing the solvent by evaporation, the crude product was purified by silica gel chromatography using ethyl acetate/hexane (2:1, v/v) as the eluent to give the desired product as a yellow solid (1.23 g, 88% yield). $^1$H NMR (500 MHz, $CDCl_3$, rt): $\delta$ 7.41 (s, 1H, ArH), 7.15 (s, 1H, ArH), 4.70-5.10 (br, 3H, CH), 3.99 (s, 1H, CH), 3.91 (d, *J* = 2.9 Hz, 2H, $CH_2$), 3.72 (s, 1H, CH), 3.36 (s, 3H, $CH_3$), 3.05 (s, 1H, OH), 2.14 (s, 1H, OH).

(a*R*)-**3**. To a solution of (a*R*)-**2** (0.68 g, 1.20 mmol), 4,7,10,13-tetraoxatetradecanoic acid (0.51 mL) and DMAP (0.33 g, 2.7 mmol) in dichloromethane (12 mL) was added EDC-HCl (0.51 g, 2.7 mmol) at 0 °C under a nitrogen atmosphere. After stirring at room temperature for 5 h, the reaction system was diluted with ethyl acetate and the solution was washed with 1 N HCl aqueous solution and water, and then dried over $Na_2SO_4$. After filtration, the solvent was removed by evaporation and the crude product was purified by silica gel chromatography using ethyl acetate as the eluent to give the desired product as a yellow viscous oil (0.54 g, 45% yield). $[\alpha]^{25}_{D}$ +167.8 (*c* 0.2, $CHCl_3$). $^1$H NMR (500 MHz, $CDCl_3$, 55 °C): $\delta$ 7.28 (s, 1H, ArH), 7.15 (s, 1H, ArH), 5.17 (s, 1H, CH), 5.09 (s, 1H, CH), 4.94 (*d*, J = 3.4 Hz, 1H, CH), 4.85 (s, 1H, CH), 4.18-4.25 (m, 2H, $CH_2$), 3.98-4.00 (m, 1H, CH), 3.76 (q, *J* = 6.9 Hz, 4H, $CH_2$), 3.59-3.63 (m, 20H, $CH_2$), 3.53 (q, *J* = 4.6 Hz, 4H, $CH_2$), 3.39 (s, 3H, $CH_3$), $3.36_3$ (s, 3H, $CH_3$), $3.36_0$ (s, 3H, $CH_3$), 2.62 (t, *J* = 6.6 Hz, 4H, $CH_2$). $^{13}$C NMR (126 MHz, $CDCl_3$, rt): $\delta$ 171.08, 170.03, 162.68, 161.56, 138.25, 136.91, 131.45, 130.27, 114.72, 97.05, 75.82, 74.26, 72.17, 70.82, 70.76, 70.69, 70.59, 70.56, 68.00, 67.79, 66.66, 66.57, 62.15,

59.05, 55.76, 35.31, 35.09. IR (KBr, $cm^{-1}$): 1742 (C=O). HRMS (FAB): *m*/*z* calcd for $C_{37}H_{51}{}^{79}Br^{81}BrO_{18}S_2$ ($M+H^+$), 1007.0858; found 1007.0894.

(a*S*)-**2**. The title compound was prepared from (a*S*)-**1** in the same way as (a*R*)-**2** and obtained in 51% yield as a yellow solid. $^1$H NMR (500 MHz, $CDCl_3$, rt): $\delta$ 7.58 (s, 1H, ArH), 7.53 (s, 1H, ArH), 4.69-4.73 (m, 2H, CH), 4.12 (dd, *J* = 10.0, 3.7 Hz, 1H, CH), 3.75-3.84 (m, 2H, $CH_2$), 3.54-3.57 (m, 1H, CH), 3.30-3.35 (m, 4H, CH, $CH_3$), 2.54 (d, *J* = 6.3 Hz, 1H, OH).

(a*S*)-**3**. The title compound was prepared from (a*S*)-**2** in the same way as (a*R*)-**3** and obtained in 73% yield as a yellow viscous oil. $[\alpha]^{25}_D$ +4.3 (*c* 0.2, $CHCl_3$). $^1$H NMR (500 MHz, $CDCl_3$, 55 °C): $\delta$ 7.53 (s, 1H, ArH), 7.51 (s, 1H, ArH), 4.82 (t, *J* = 9.7 Hz, 1H, CH), 4.71 (d, *J* = 3.4 Hz, 1H, CH), 4.66 (t, *J* = 9.7 Hz, 1H, CH), 4.10-4.13 (m, 3H, CH, $CH_2$), 3.82-3.85 (m, 1H, CH), 3.74 (t, *J* = 6.6 Hz, 2H, $CH_2$), 3.49-3.65 (m, 26H, $CH_2$), $3.37_3$ (s, 3H, $CH_3$), $3.36_5$ (s, 3H, $CH_3$), 3.31 (s, 3H, $CH_3$), 2.62 (t, *J* = 6.6 Hz, 2H, $CH_2$), 2.40-2.50 (m, 2H, $CH_2$). $^{13}$C NMR (151 MHz, $CDCl_3$, 55 °C): $\delta$ 171.04, 170.12, 159.66, 158.90, 141.22, 140.12, 132.51, 132.43, 132.31, 132.23, 113.66, 113.16, 96.31, 96.21, 72.24, 70.92, 70.88, 70.86, 70.82, 70.75, 70.65, 70.58, 70.48, 69.74, 69.67, 69.62, 69.46, 69.41, 67.25, 67.15, 66.74, 66.66, 66.56, 66.52, 66.46, 66.39, 62.19, 62.11, 62.01, 59.12, 55.36, 35.15, 35.10, 35.03. IR (KBr, $cm^{-1}$): 1746 (C=O), 1725 (C=O). HRMS (FAB): *m*/*z* calcd for $C_{37}H_{51}{}^{79}Br^{81}BrO_{18}S_2$ ($M+H^+$), 1007.0858; found 1007.0842.

**6a**. To a solution of 5,5′-dibromo-2,2′-bithiophene-3,3′-dicarboxylic acid (120 mg, 0.29 mmol), tetraethylene glycol monomethyl ether (0.11 mL, 0.56 mmol) and DMAP (78 mg, 0.64 mmol) in dichloromethane (3 mL) was added EDC-HCl (123 mg, 0.64 mmol) at 0 °C under a nitrogen atmosphere. After stirring at room temperature for 16 h, the reaction system was diluted with ethyl acetate and the solution was washed with 1 N HCl aqueous solution and water, and then dried over $Na_2SO_4$. After filtration, the solvent was removed by evaporation and the crude product was purified by silica gel chromatography using ethyl acetate as the eluent to give the desired product as a colorless viscous oil (80 mg, 35% yield). $^1$H NMR (600 MHz, $CDCl_3$, 55 °C): $\delta$ 7.49 (s, 2H, ArH), 4.23 (t, *J* = 5.0 Hz, 4H, $CH_2$), 3.52-3.63 (m, 28H, $CH_2$), 3.38 (s, 6H, $CH_3$). $^{13}$C NMR (151 MHz, $CDCl_3$, 55 °C): $\delta$ 161.20, 139.87, 132.68, 132.17, 113.52, 72.25, 70.92, 70.89, 70.84, 70.77, 69.04, 64.36, 59.12. IR (KBr, $cm^{-1}$): 1720 (C=O). HRMS (FAB): *m*/*z* calcd for $C_{28}H_{41}{}^{79}Br^{81}BrO_{12}S_2$ ($M^+$), 793.0380; found 793.0384.

**6b**-prec-2. To a solution of **6b**-prec-1 (300 mg, 0.454 mmol) in acetonitrile/water (300:1, v/v) (12 mL) was added $HClO_4$–$SiO_2$ (400 mg). After stirring at room temperature for 1 h, the reaction

mixture was diluted with ethyl acetate and passed through a short pad of silica gel using ethyl acetate as the eluent to remove $HClO_4$–$SiO_2$. After concentrating in vacuo, the crude product was passed through a short pad of silica gel using ethyl acetate/hexane (2:1, v/v) as the eluent and the solvent was removed under a reduced pressure to give the crude **6b**-prec-2 as a colorless viscous oil (151 mg), which was used for the next step without further purification.

**6b**. To a solution of **6b**-prec-2 (151 mg), 4,7,10,13-tetraoxatetradecanoic acid (0.14 mL, 0.58 mmol) and DMAP (71 mg, 0.58 mmol) in dichloromethane (3 mL) was added EDC-HCl (111 mg, 0.58 mmol) at 0 °C under a nitrogen atmosphere. After stirring at room temperature for 16 h, the reaction system was diluted with ethyl acetate and the solution was washed with 1 N HCl aqueous solution and water, and then dried over $Na_2SO_4$. After filtration, the solvent was removed by evaporation and the crude product was purified by silica gel chromatography using ethyl acetate as the eluent to give the desired product as a colorless viscous oil (171 mg, 37% yield over 2 steps). $[\alpha]^{25}_{D}$ +77.5 (*c* 0.2, $CHCl_3$). $^{1}H$ NMR (600 MHz, $CDCl_3$, 55 °C): $\delta$ 7.94 (d, *J* = 25.8 Hz, 2H, ArH), 7.38 (d, *J* = 10.0 Hz, 2H, ArH), 5.74 (t, *J* = 9.8 Hz, 1H, CH), 5.25 (t, *J* = 9.8 Hz, 1H, CH), 5.09 (d, *J* = 3.8 Hz, 1H, CH), 5.03 (dd, *J* = 10.1, 3.6 Hz, 1H, CH), 4.21-4.29 (m, 2H, $CH_2$), 4.06-4.09 (m, 1H, CH), 3.77 (t, *J* = 6.5 Hz, 2H, $CH_2$), 3.58-3.64 (m, 18H, $CH_2$), 3.52-3.54 (m, 4H, $CH_2$), 3.44-3.49 (m, 4H, $CH_2$), 3.43 (s, 3H, $CH_3$), 3.37 (s, 6H, $CH_3$), 2.65 (t, *J* = 6.5 Hz, 2H, $CH_2$), 2.45-2.54 (m, 2H, $CH_2$). $^{13}C$ NMR (126 MHz, $CDCl_3$, 55 °C): $\delta$ 171.17, 170.31, 160.62, 135.31, 135.00, 133.07, 132.93, 130.52, 130.48, 130.44, 130.40, 113.38, 113.35, 97.28, 97.14, 72.25, 72.14, 71.08, 71.03, 70.96, 70.90, 70.84, 70.78, 70.67, 70.53, 68.60, 68.51, 67.80, 67.72, 66.75, 66.68, 66.59, 66.52, 66.46, 62.39, 62.33, 62.27, 59.11, 55.79, 35.32, 35.27, 35.23, 35.16. IR (KBr, $cm^{-1}$): 1731 (C=O). HRMS (FAB): *m*/*z* calcd for $C_{37}H_{52}{}^{79}Br^{81}BrNaO_{18}S_2$ ($M+Na^+$), 1031.0834; found 1031.0816.

model-$T_R$. To a solution of 2-(tributylstannyl)thiophene (**4b**) (35 mg, 0.094 mmol) and (a*R*)-**3** (43 mg, 0.043 mmol) in anhydrous toluene/DMF (4:1, v/v) (2.0 mL) was added $Pd(PPh_3)_4$ (4.0 mg, 3.4 μmol). The solution was stirred at 110 °C for 12 h. After cooling to room temperature, the reaction system was diluted with ethyl acetate/hexane (3:1, v/v) and the solution was washed with water, and then dried over $Na_2SO_4$. After filtration, the solvent was removed by evaporation and the crude product was purified by silica gel chromatography using ethyl acetate/methanol (20:1, v/v) as the eluent to give the desired product as a yellow viscous oil (25 mg, 57% yield). $[\alpha]^{25}_{D}$ +287.0 (*c* 0.2, $CHCl_3$). $^{1}H$ NMR (500 MHz, $CDCl_3$, 55 °C): $\delta$ 7.35 (s, 1H, ArH), 7.31 (t, *J* = 4.0 Hz, 2H, ArH), 7.23-7.26 (m, 3H, ArH), 7.06 (q, *J* = 4.8 Hz, 2H, ArH), 5.18 (s, 2H, CH), 4.97 (d, *J* = 3.4 Hz, 1H, CH), 4.92 (s, 1H, CH), 4.18-4.25 (m, 2H, $CH_2$), 3.99 (s, 1H, CH), 3.79 (t, *J* = 6.0 Hz, 2H, $CH_2$),

3.75 (t, $J = 6.6$ Hz, 2H, $CH_2$), 3.55-3.63 (m, 20H, $CH_2$), 3.50-3.54 (m, 4H, $CH_2$), 3.39 (s, 3H, $CH_3$), 3.36 (s, 3H, $CH_3$), 3.35 (s, 3H, $CH_3$), 2.61-2.66 (m, 4H, $CH_2$). $^{13}$C NMR (126 MHz, $CDCl_3$, 55 °C): $\delta$ 171.16, 170.07, 138.99, 135.49, 135.32, 128.37, 128.32, 126.35, 126.25, 125.50, 125.45, 97.22, 72.24, 72.21, 70.87, 70.80, 70.76, 70.70, 70.65, 68.27, 67.78, 66.73, 66.64, 62.29, 59.09, 55.78, 35.40, 35.14. IR (KBr, cm$^{-1}$): 1742 (C=O). HRMS (FAB): *m*/*z* calcd for $C_{45}H_{56}O_{18}S_4$ ($M^+$), 1012.2344; found 1012.2366.

model-Ph$_R$. The title compound was prepared from (a*R*)-**3** and trimethyl(phenyl)tin (**5b**) in the same way as model-T$_R$ and obtained in 43% yield as a yellow viscous oil. $[\alpha]^{25}_D$ +182.5 (*c* 0.2, $CHCl_3$). $^1$H NMR (500 MHz, $CDCl_3$, 55 °C): $\delta$ 7.61 (dd, $J = 12.9$, 7.7 Hz, 4H, ArH), 7.35-7.45 (m, 8H, ArH), 5.19 (s, 2H, CH), 4.97 (s, 1H, CH), 4.94 (s, 1H, CH), 4.18-4.25 (m, 2H, $CH_2$), 3.99 (s, 1H, CH), 3.80 (t, $J = 6.0$ Hz, 2H, $CH_2$), 3.74 (t, $J = 6.6$ Hz, 2H, $CH_2$), 3.49-3.63 (m, 24H, $CH_2$), 3.34-3.38 (m, 9H, $CH_3$), 2.61-2.66 (m, 4H, $CH_2$). $^{13}$C NMR (126 MHz, $CDCl_3$, 55 °C): $\delta$ 171.16, 170.10, 145.79, 132.92, 132.82, 129.42, 129.39, 128.97, 126.23, 126.19, 97.30, 97.24, 72.21, 70.87, 70.76, 70.65, 68.35, 67.78, 66.74, 66.63, 62.31, 59.09, 55.77, 35.41, 35.14. IR (KBr, cm$^{-1}$): 1741 (C=O). HRMS (FAB): *m*/*z* calcd for $C_{49}H_{61}O_{18}S_2$ (M+H$^+$), 1001.3294; found 1001.3290.

di-model-T$_R$ (Inseparable mixture of model-A/model-B). To a solution of (a*R*)-**3** (70 mg, 0.069 mmol) and 2,5-bis(trimethylstannyl)thiophene (**4**) (7.1 mg, 0.017 mmol) in anhydrous toluene/DMF (4:1, v/v) (2.0 mL) was added $Pd(PPh_3)_4$ (6.4 mg, 5.5 μmol). The solution was stirred at 110 °C for 24 h. After cooling to room temperature, the reaction system was diluted with ethyl acetate/hexane (3:1, v/v) and the solution was washed with water, and then dried over $Na_2SO_4$. After filtration, the solvent was removed by evaporation and the crude product was purified by silica gel chromatography using dichloromethane/methanol (10:1, v/v) as the eluent to give the desired model-A/model-B mixture as an orange viscous oil (13 mg, 10% yield). $[\alpha]^{25}_D$ +283.0 (*c* 0.2, $CHCl_3$). $^1$H NMR (500 MHz, $CDCl_3$, 55 °C): $\delta$ 7.17-7.58 (m, 6H, ArH), 5.10-5.30 (br, 4H, CH), 4.80-5.00 (br, 4H, CH), 4.19-4.26 (m, 4H, $CH_2$), 4.01 (s, 2H, CH), 3.74-3.79 (m, 8H, $CH_2$), 3.61 (m, 40H, $CH_2$), 3.52 (m, 8H, $CH_2$), 3.41 (s, 6H, $CH_3$), 3.35 (m, 12H, $CH_3$), 2.63 (t, $J = 6.3$ Hz, 8H, $CH_2$). IR (KBr, cm$^{-1}$): 1741 (C=O). HRMS (FAB): *m*/*z* calcd for $C_{78}H_{103}{}^{79}Br^{81}BrO_{36}S_5$ (M+H$^+$), 1935.3173; found 1935.3144.

poly-T$_R$. Copolymerization of (a*R*)-**3** with **4** by Stille cross-coupling was carried out in a dry Schlenk flask under nitrogen atmosphere. To a solution of (a*R*)-**3** (55 mg, 0.054 mmol) and **4** (22 mg, 0.054 mmol) in anhydrous toluene/DMF (4:1, v/v) (1.1 mL) was added $Pd(PPh_3)_4$ (5.0 mg, 4.3

μmol). The solution was stirred at 110 °C for 24 h. After cooling to room temperature, the reaction mixture was poured into a large amount of hexane, and the resulting polymer was collected by centrifugation, washed with ethanol and dried *in vacuo* to yield poly-$T_R$ as an orange solid (46 mg, 91%). $^1$H NMR (500 MHz, $CDCl_3$, 55 °C): $\delta$ 7.00-7.80 (br, 4H, ArH), 4.60-5.50 (br, 4H, CH), 3.90-4.50 (br, 3H, CH, $CH_2$), 3.20-3.90 (m, 37H, $CH_2$, $CH_3$), 2.40-2.90 (br, 4H, $CH_2$). IR (KBr, $cm^{-1}$): 1740 (C=O).

poly-$T_R'$. The title compound was prepared from (a*R*)-**3**, **6a** and **4** ([(a*R*)-**3**]$_0$/[**6a**]$_0$/[**4**]$_0$ = 1/1/2) in the same way as poly-$T_R$ and obtained in 92% yield as an orange solid. $^1$H NMR (500 MHz, $CDCl_3$, 55 °C): $\delta$ 7.50-7.70 (br, 2H, ArH), 7.10-7.30 (br, 2H, ArH), 4.70-5.50 (br, 2H, CH), 3.90-4.40 (br, 3.5H, CH, $CH_2$), 3.20-3.90 (m, 35.5H, $CH_2$, $CH_3$), 2.40-2.70 (br, 2H, $CH_2$). IR (KBr, $cm^{-1}$): 1719 (C=O).

poly-$T_R''$. The title compound was prepared from (a*R*)-**3**, **6b** and **4** ([(a*R*)-**3**]$_0$/[**6b**]$_0$/[**4**]$_0$ = 1/1/2) in the same way as poly-$T_R$ and obtained in 89% yield as an orange solid. $^1$H NMR (500 MHz, $CDCl_3$, 55 °C): $\delta$ 7.80-8.10 (m, 1H, ArH), 7.40-7.70 (m, 2H, ArH), 6.90-7.30 (m, 2H, ArH), 4.50-5.90 (br, 4H, CH), 3.90-4.50 (br, 3H, CH, $CH_2$), 3.20-3.90 (br, 37H, $CH_2$, $CH_3$), 2.40-2.70 (br, 4H, $CH_2$). IR (KBr, $cm^{-1}$): 1735 (C=O).

poly-$Ph_R$. The title compound was prepared from (a*R*)-**3** and 1,4-bis(tributylstannyl)benzene (**5**) in the same way as poly-$T_R$ and obtained in 57% yield as a yellow solid. $^1$H NMR (500 MHz, $CDCl_3$, 55 °C): $\delta$ 7.30-7.80 (m, 6H, ArH), 5.10-5.40 (br, 2H, CH), 4.70-5.10 (br, 2H, CH), 4.10-4.30 (br, 2H, $CH_2$), 3.90-4.10 (br, 1H, CH), 3.34-3.82 (m, 37H, $CH_2$, $CH_3$), 2.50-2.70 (br, 4H, $CH_2$). IR (KBr, $cm^{-1}$): 1741 (C=O).

## 4. All-atom molecular dynamics simulation

An all-atom molecular dynamics (MD) simulation was carried out using the Forcite module of the BIOVIA Materials Studio 2018 (Dassault Systèmes BIOVIA, San Diego, CA, USA) on the supercomputer system (PRIMERGY CX2570 M4, Fujitsu, Tokyo, Japan). The MD cell was built by means of usual procedure of the Amorphous Cell module. The MD cell length and angle were (a = 80 Å, b = 80 Å, c = 80 Å) and (α = 90º, β = 90º, γ = 90º), respectively. Here, a single model of poly-$T_R$ was put in the center of the cell, and the solvent molecules of chloroform were packed in the cell at density of 1.492 g $cm^{-3}$. Sequentially, the geometry of the MD cell was optimized. Simulation in the NVT ensemble (constant number of atoms, volume and temperature) was conducted at 298 K for 20 ps (time step of 0.2-fs, 100,000 steps) and the NPT ensemble (constant number of atoms, pressure and temperature) was conducted at pressure of $1.013 \times 10^{-4}$ GPa and at 298 K for 3,000 ps (time step of 1.0-fs, 3,000,000 steps) to equilibrate the MD cell. The Nose thermostat was used to control the temperature. The Berendsen barostat was used to control the pressure. After the equilibration at 298 K, simulation in the NVE ensemble (constant number of atoms, volume and energy) was conducted for 2,000 ps (time step of 1.0-fs, 1,000,000 steps) as the production run. The Universal forcefield was used, and the charges were assigned by the Gasteiger.

## Supporting data

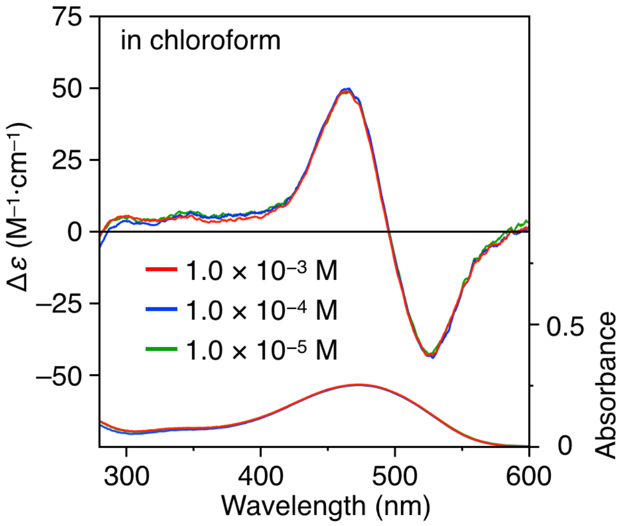


**Fig. S1** Concentration dependence of the absorption and CD spectra of poly-T$_R$ in chloroform at 25 °C. Spectra indicated by green, blue and red lines were obtained from solutions with poly-T$_R$ concentrations of $1.0 \times 10^{-5}$ M (cell length, 10 mm), $1.0 \times 10^{-4}$ M (cell length, 1.0 mm) and $1.0 \times 10^{-3}$ M (cell length, 0.10 mm), respectively.

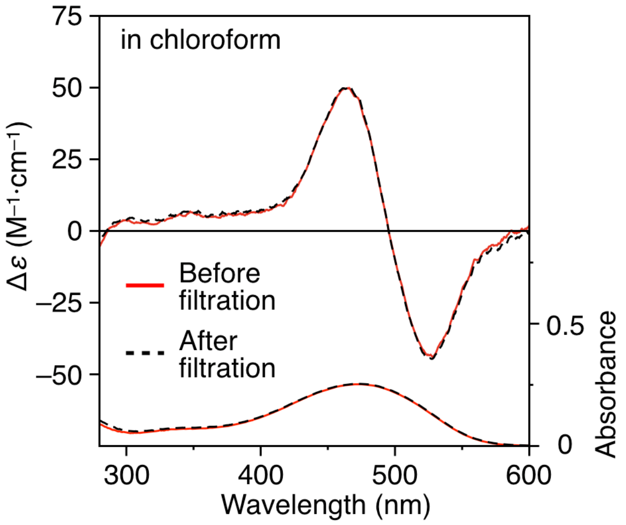


**Fig. S2** Absorption and CD spectra of poly-T$_R$ in chloroform at 25 °C before (red solid line) and after (black dashed line) filtration through a membrane filter with a pore size of 0.20 μm. [Glucose unit] = $1.0 \times 10^{-4}$ M.

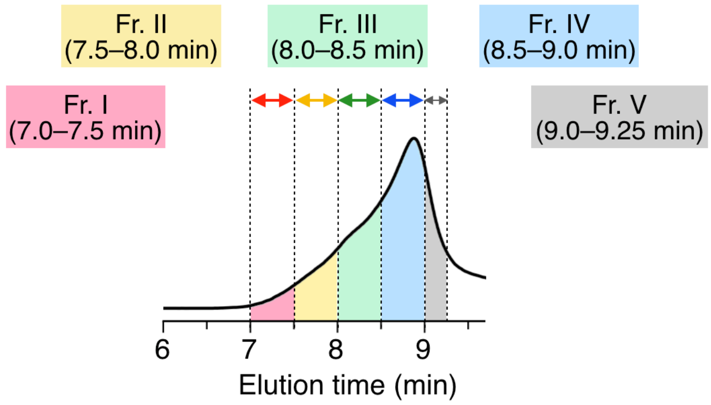


**Fig. S3** SEC trace of as-synthesized poly-T$_R$ ($M_n$: 6.7 × $10^3$ g $mol^{-1}$, $M_w/M_n$: 2.3). Fractionation intervals are also shown.

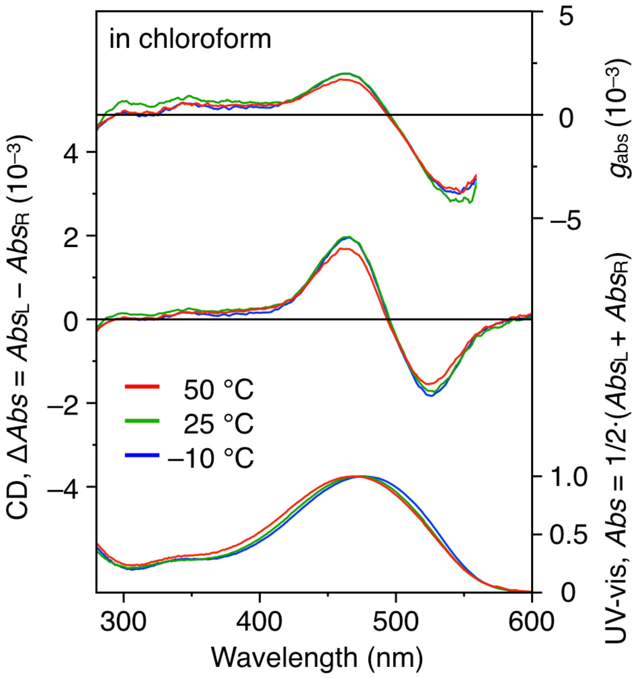


**Fig. S4** Temperature dependence of the absorption (bottom), CD (middle) and $g_{abs}$ (top) spectra of poly-T$_R$ in chloroform. [Glucose unit] = 1.0 × $10^{-4}$ M.

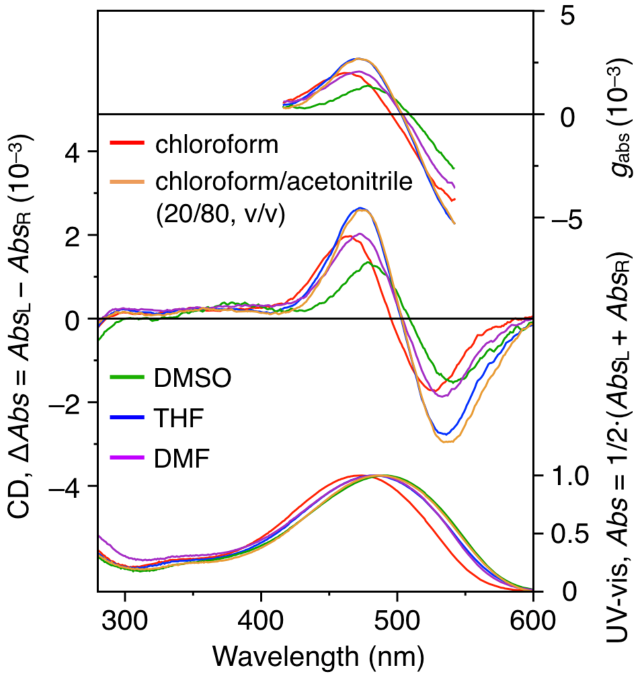


**Fig. S5** Absorption (bottom), CD (middle) and $g_{abs}$ (top) spectra of poly-$T_R$ in different solvents at 25 °C. [Glucose unit] = 1.0 × $10^{-4}$ M.

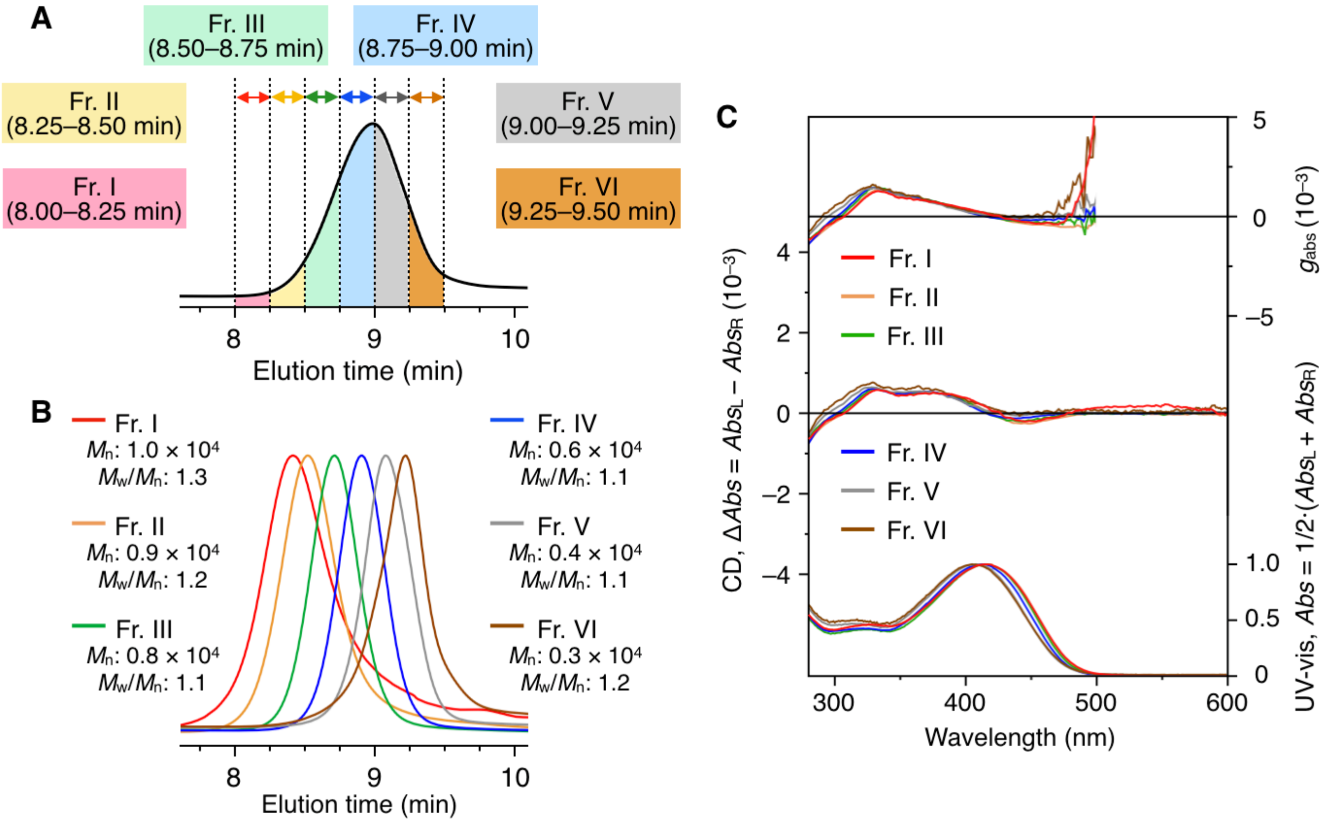


**Fig. S6** (A) SEC trace of as-synthesized poly-Ph$_R$ ($M_n$: 5.6 × 10$^3$ g mol$^{-1}$, $M_w/M_n$: 1.2). Fractionation intervals are also shown. (B) SEC traces of fractionated components of poly-Ph$_R$ with different molecular masses (eluent, chloroform; polystyrene standards). (C) Molecular mass dependence of the CD and absorption spectra of poly-Ph$_R$ in chloroform at 25 °C.

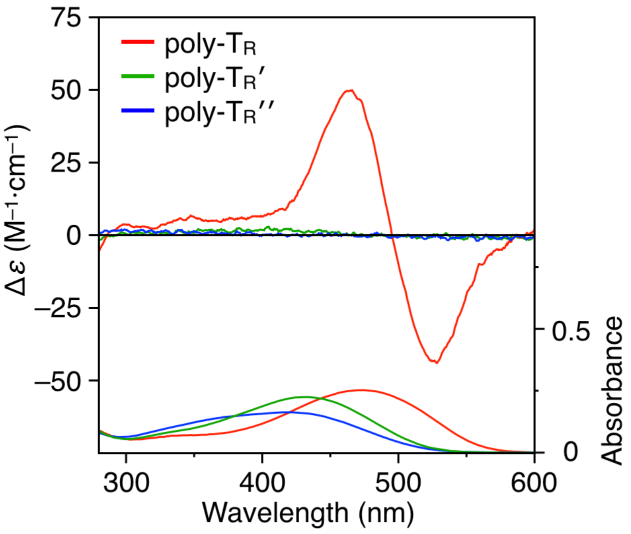


**Fig. S7** CD and absorption spectra of poly-T$_R$, poly-T$_R$′ and poly-T$_R$″ in chloroform at 25 °C. [3,4-Unsbstituted thiophene unit] = 1.0 × 10$^{-4}$ M.

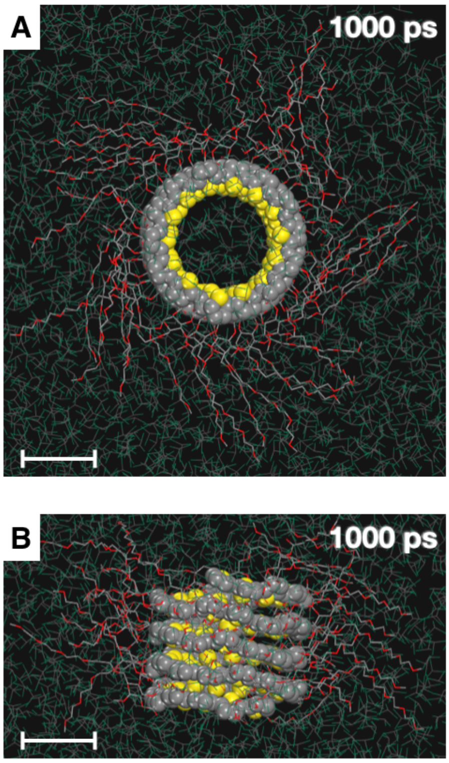


**Fig. S8** (A) Top view and (B) side view of the molecular model of the left-handed helically folded poly-$T_R$ in chloroform at 1000 ps in an all-atom MD simulation after equilibration at 298 K, represented by space-filling (polythiophene backbone) and stick (side chain) models. Chloroform solvent molecules are represented by line models and their hydrogen atoms are omitted to simplify the view. Scale bars represent 1 nm.

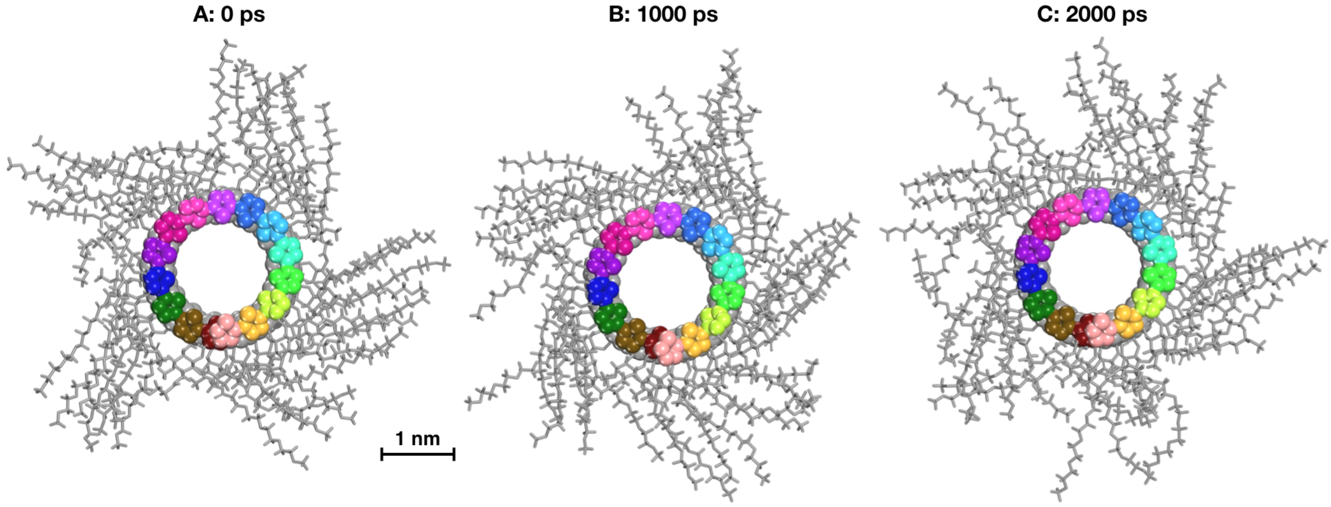


**Fig. S9** Top view of the molecular model of the left-handed helically folded poly-$T_R$ in chloroform at (A) 0 ps, (B) 1000 ps and (C) 2000 ps in an all-atom MD simulation after equilibration at 298 K, represented by space-filling (polythiophene backbone) and stick (side chain) models. The first 15 thiophene rings, counting from the top of the model, are shown in different colors; other repeating units are shown in grey to clarify the number of thiophene units contained in a single helix turn. Chloroform solvent molecules are omitted to simplify the view.

**Table S1.** Average torsion angles ($\theta_i$) and standard deviations (SD) of the left-handed helically folded poly-$T_R$ model [a]

| $\theta_i$ | Average torsion angle (deg)[b] | SD[c] |
|---|---|---|
| $\theta_2$ | −50.7 | 8.74 |
| $\theta_3$ | −46.3 | 8.41 |
| $\theta_4$ | −47.7 | 8.15 |
| $\theta_5$ | −46.9 | 7.12 |
| $\theta_6$ | −42.6 | 7.62 |
| $\theta_7$ | −42.6 | 7.56 |
| $\theta_8$ | −44.7 | 8.79 |
| $\theta_9$ | −45.3 | 7.23 |
| $\theta_{10}$ | −43.0 | 8.05 |
| $\theta_{11}$ | −44.2 | 8.17 |
| $\theta_{12}$ | −40.7 | 8.09 |
| $\theta_{13}$ | −40.0 | 7.45 |
| $\theta_{14}$ | −41.6 | 8.24 |
| $\theta_{15}$ | −41.8 | 7.54 |
| $\theta_{16}$ | −40.9 | 7.60 |
| $\theta_{17}$ | −42.0 | 7.89 |
| $\theta_{18}$ | −41.8 | 8.24 |
| $\theta_{19}$ | −42.9 | 8.21 |

[a]Simulation results after equilibration at 298 K. [b]Average value during the whole calculation period (0–2000 ps). [c]The number of samples is 200.

**Table S2.** Average torsion angles ($\phi_i$) and SD values of the left-handed helically folded poly-$T_R$ model[a]

| $\phi_i$ | Average torsion angle (deg)[b] | SD[c] |
|---|---|---|
| $\phi_2$ | +21.1 | 9.70 |
| $\phi_3$ | +18.9 | 9.81 |
| $\phi_4$ | +19.0 | 9.92 |
| $\phi_5$ | +23.1 | 8.54 |
| $\phi_6$ | +18.4 | 9.07 |
| $\phi_7$ | +18.9 | 10.67 |
| $\phi_8$ | +16.7 | 9.44 |
| $\phi_9$ | +18.0 | 9.75 |
| $\phi_{10}$ | +17.7 | 10.07 |
| $\phi_{11}$ | +17.6 | 9.91 |
| $\phi_{12}$ | +15.9 | 9.74 |
| $\phi_{13}$ | +15.9 | 9.71 |
| $\phi_{14}$ | +14.7 | 10.22 |
| $\phi_{15}$ | +17.0 | 10.87 |
| $\phi_{16}$ | +14.4 | 9.90 |
| $\phi_{17}$ | +14.1 | 10.84 |
| $\phi_{18}$ | +12.5 | 10.23 |
| $\phi_{19}$ | +11.0 | 10.51 |

[a]Simulation results after equilibration at 298 K. [b]Average value during the whole calculation period (0–2000 ps). [c]The number of samples is 200.

**Table S3.** Average torsion angles ($\psi_i$) and SD values of the left-handed helically folded poly-$T_R$ model [a]

| $\psi_i$ | Average torsion angle (deg)[b] | SD[c] |
|---|---|---|
| $\psi_2$ | +19.7 | 10.13 |
| $\psi_3$ | +18.0 | 10.36 |
| $\psi_4$ | +17.0 | 10.74 |
| $\psi_5$ | +14.7 | 9.55 |
| $\psi_6$ | +16.4 | 9.95 |
| $\psi_7$ | +16.1 | 10.36 |
| $\psi_8$ | +18.2 | 9.87 |
| $\psi_9$ | +17.3 | 9.66 |
| $\psi_{10}$ | +16.5 | 9.88 |
| $\psi_{11}$ | +18.1 | 10.03 |
| $\psi_{12}$ | +16.2 | 10.17 |
| $\psi_{13}$ | +17.5 | 9.63 |
| $\psi_{14}$ | +17.7 | 10.47 |
| $\psi_{15}$ | +18.2 | 10.62 |
| $\psi_{16}$ | +22.3 | 9.29 |
| $\psi_{17}$ | +20.6 | 9.62 |
| $\psi_{18}$ | +20.5 | 10.43 |
| $\psi_{19}$ | +21.1 | 10.32 |

[a]Simulation results after equilibration at 298 K. [b]Average value during the whole calculation period (0–2000 ps). [c]The number of samples is 200.

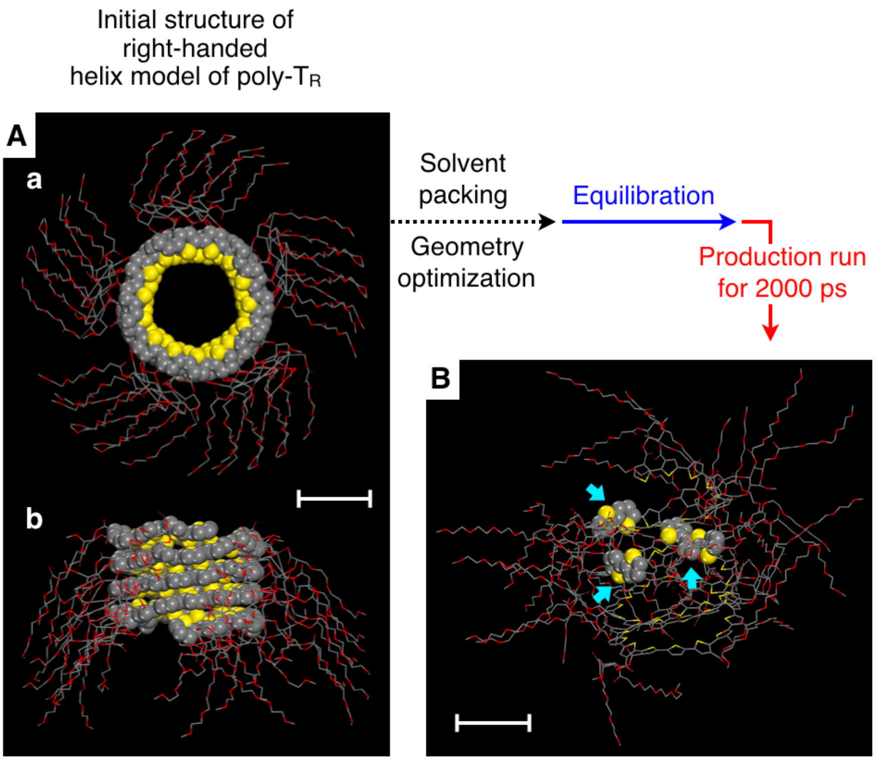


**Fig. S10** (A) (a) Top view and (b) side view of the initial structure of right-handed helix model of poly-$T_R$, represented by space-filling (polythiophene backbone) and stick (side chain) models. (B) Side view of the right-handed helically folded poly-$T_R$ in chloroform at 2000 ps in an all-atom MD simulation after equilibration at 298 K. Thiophene sequences with *anti*-conformations (blue arrows) are represented by space-filling models; other parts are represented by stick models to clarify the structural irregularity emerging in the main chain. Chloroform solvent molecules are omitted to simplify the view.

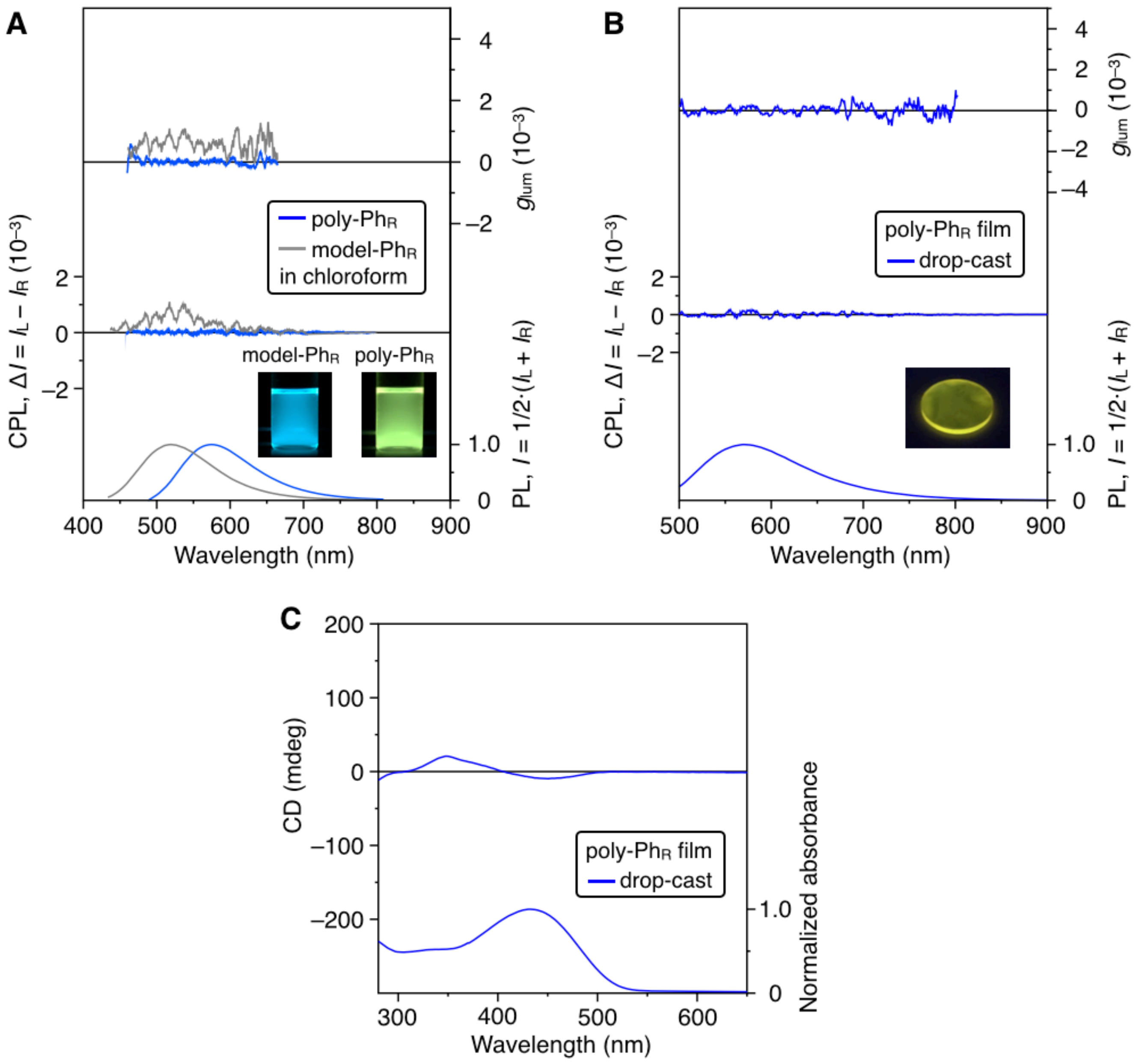


**Fig. S11** PL (bottom), CPL (middle) and $g_{lum}$ (top) spectra of poly-Ph$_R$ and model-Ph$_R$ (A) in chloroform ([Glucose unit] = 1.0 × 10$^{-4}$ M) and (B) the drop-cast film of poly-Ph$_R$ at room temperature. $\lambda_{ex}$ = 365 nm. Insets: Photographs of the corresponding solutions and film under irradiation at 365 nm. (C) Absorption and CD spectra of the drop-cast film of poly-Ph$_R$ at room temperature.

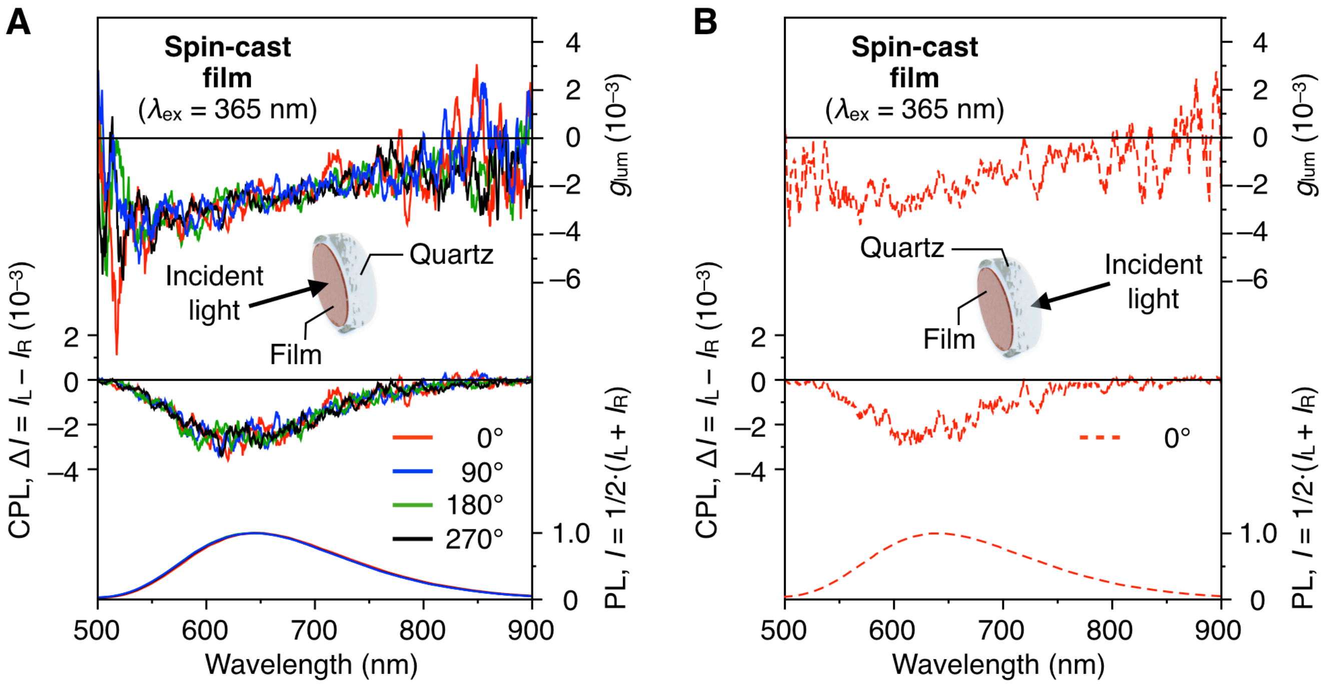


**Fig. S12** PL (bottom), CPL (middle) and $g_{\mathrm{lum}}$ (top) spectra of the spin-cast film of poly-$T_R$ at room temperature. Spectra were measured (A) at different rotation angles and (B) by reversing the quartz plate to an incident light/quartz/film arrangement. $\lambda_{ex}$ = 365 nm.

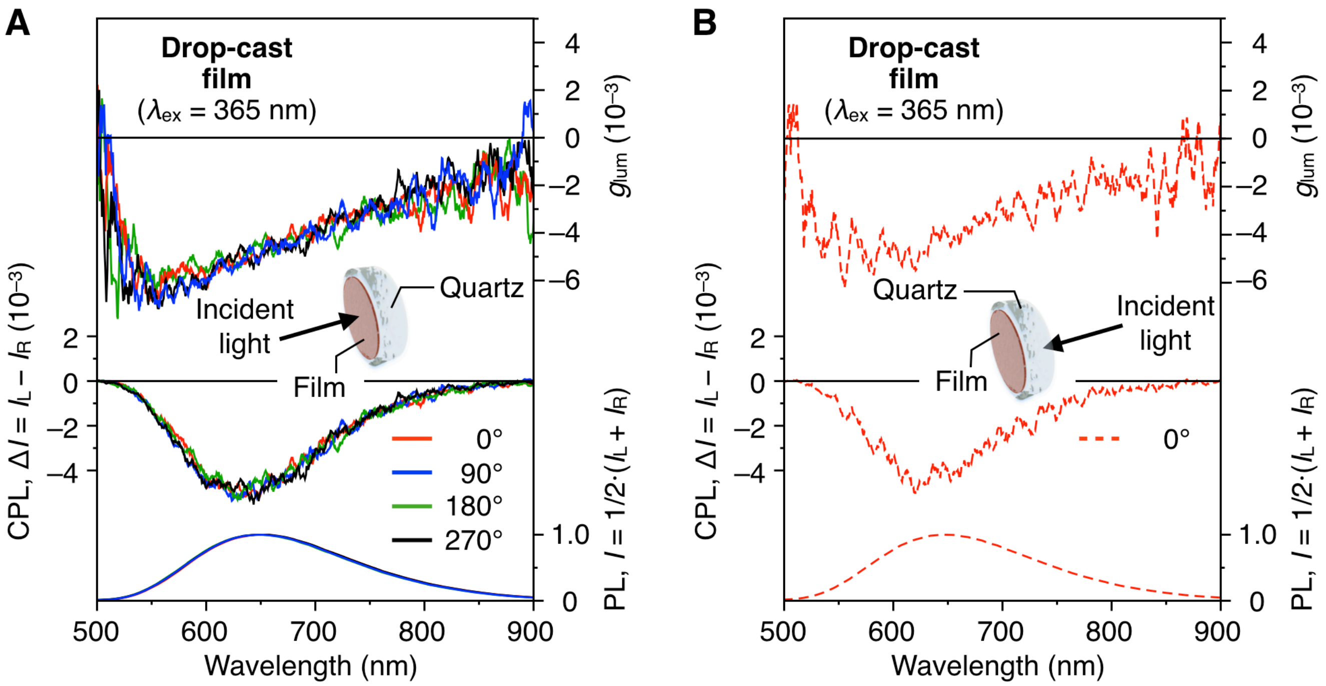


**Fig. S13** PL (bottom), CPL (middle) and $g_{\mathrm{lum}}$ (top) spectra of the drop-cast film of poly-$T_R$ at room temperature. Spectra were measured (A) at different rotation angles and (B) by reversing the quartz plate to an incident light/quartz/film arrangement. $\lambda_{ex}$ = 365 nm.

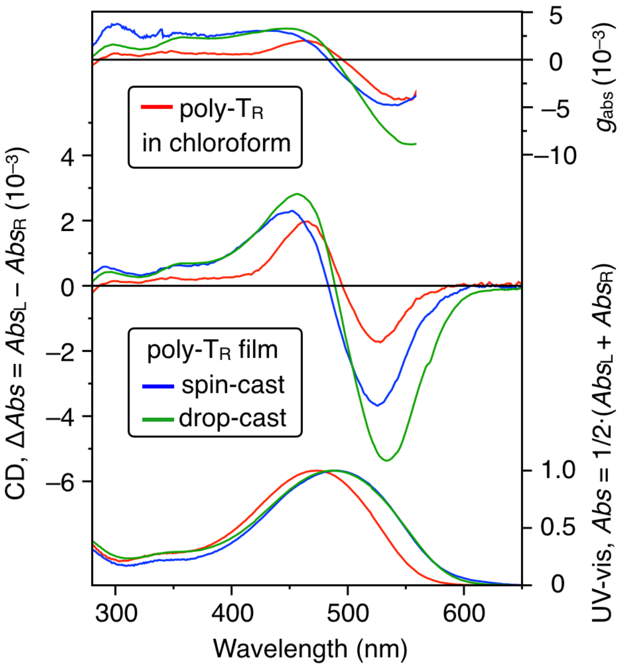


**Fig. S14** Absorption (bottom), CD (middle) and $g_{abs}$ (top) spectra of poly-$T_R$ in chloroform at 25 °C ([Glucose unit] = $1.0 \times 10^{-4}$ M) and its spin- and drop-cast films (room temperature).

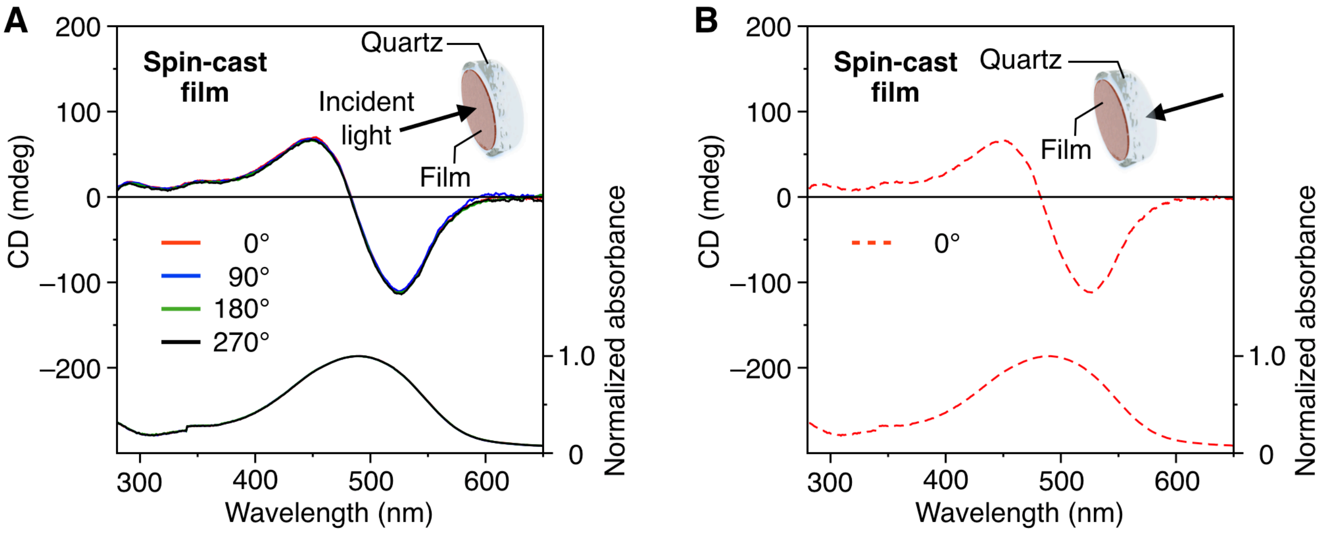


**Fig. S15** CD and absorption spectra of the spin-cast film of poly-T$_R$ at room temperature. Spectra were measured at (A) different rotation angles and (B) by reversing the quartz plate to an incident light/quartz/film arrangement.

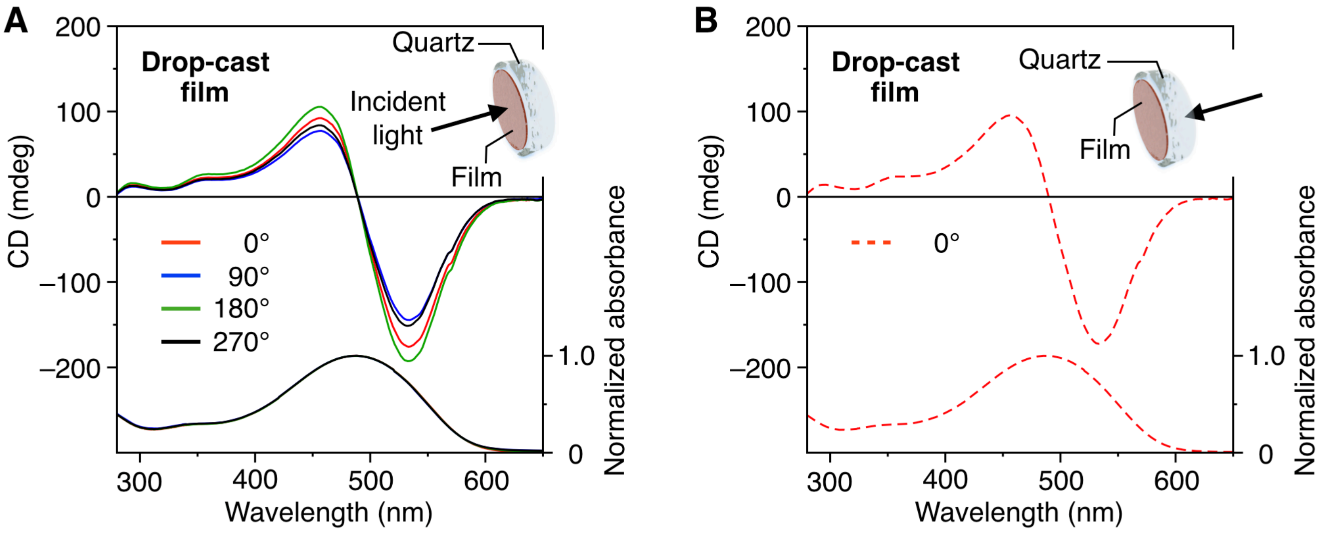


**Fig. S16** CD and absorption spectra of the drop-cast film of poly-T$_R$ at room temperature. Spectra were measured at (A) different rotation angles and (B) by reversing the quartz plate to an incident light/quartz/film arrangement.

## NMR spectral data

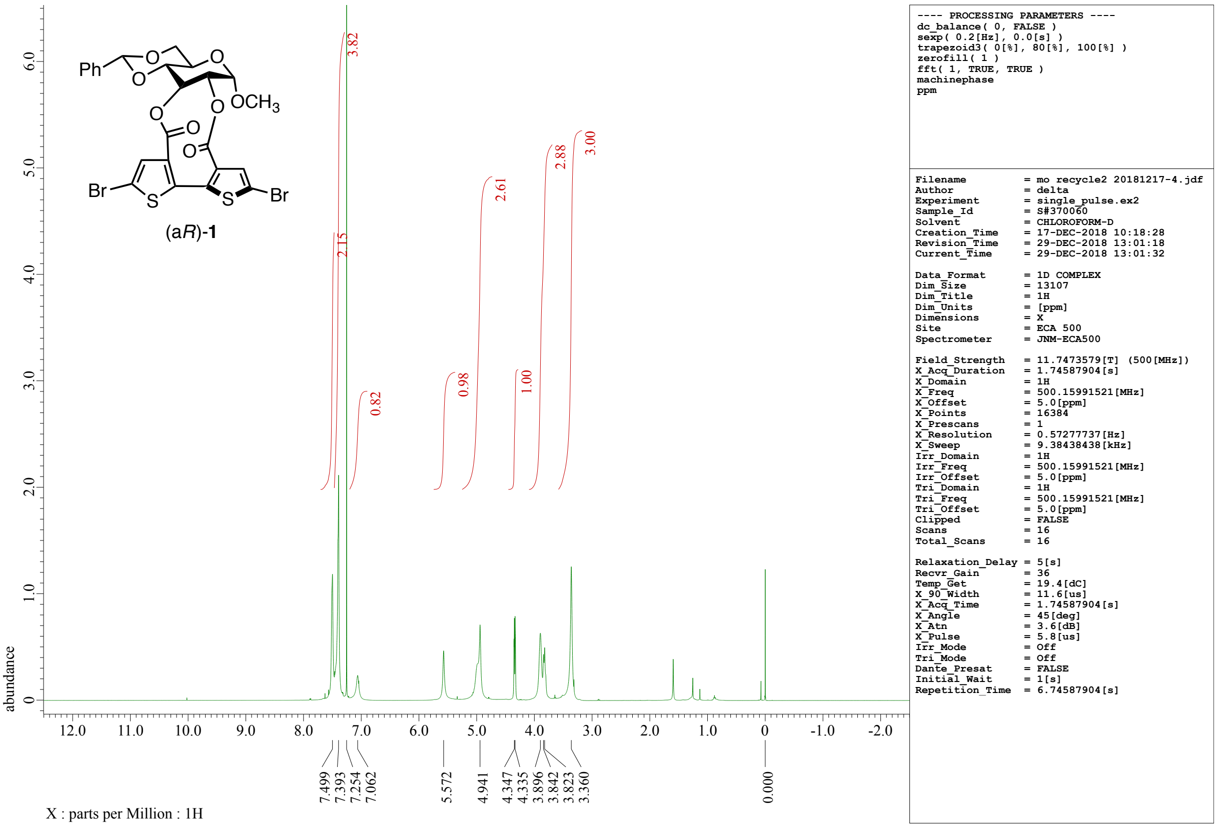


**Fig. S17** $^{1}$H NMR (500 MHz, $CDCl_3$, rt) spectrum of (a*R*)-**1**.

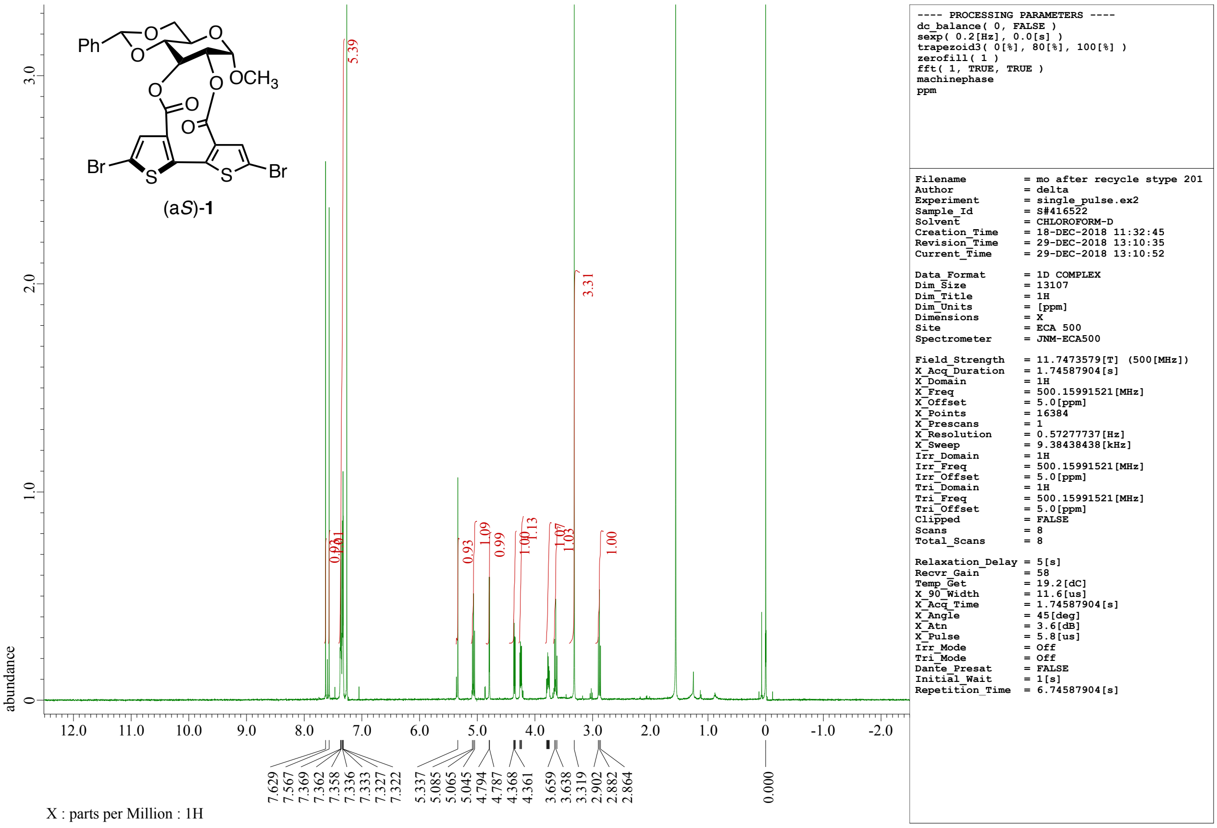


**Fig. S18** $^{1}$H NMR (500 MHz, $CDCl_3$, rt) spectrum of (a*S*)-**1**.

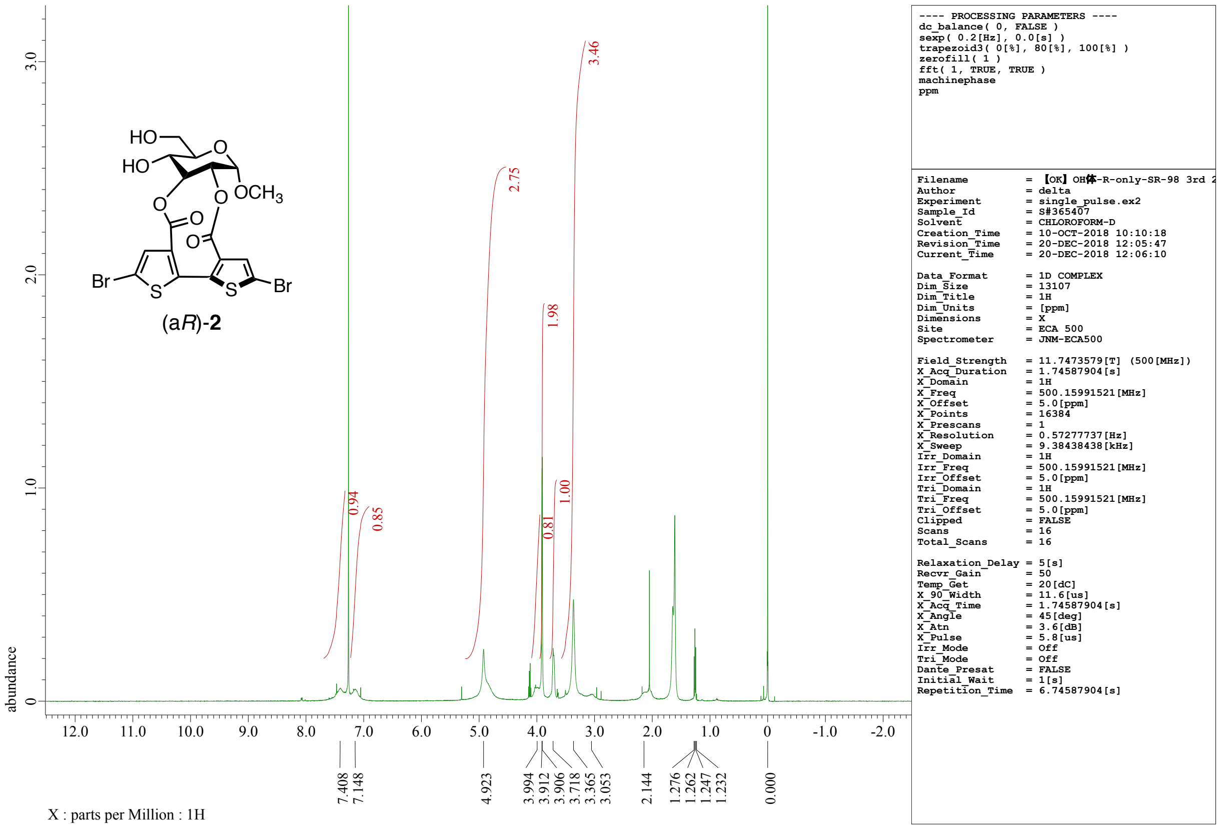


**Fig. S19** $^{1}$H NMR (500 MHz, $CDCl_3$, rt) spectrum of (a*R*)-**2**.

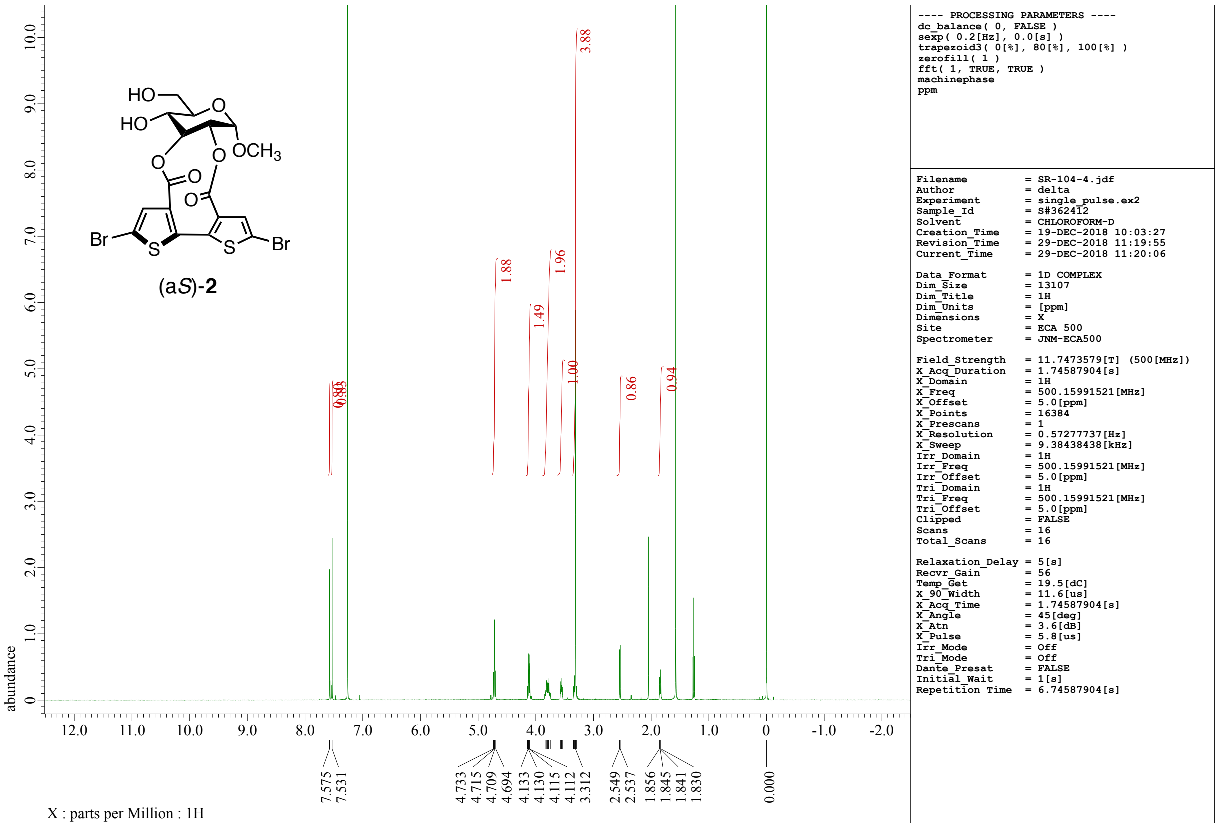


**Fig. S20** $^{1}$H NMR (500 MHz, $CDCl_3$, 55 °C) spectrum of (a*S*)-**2**.

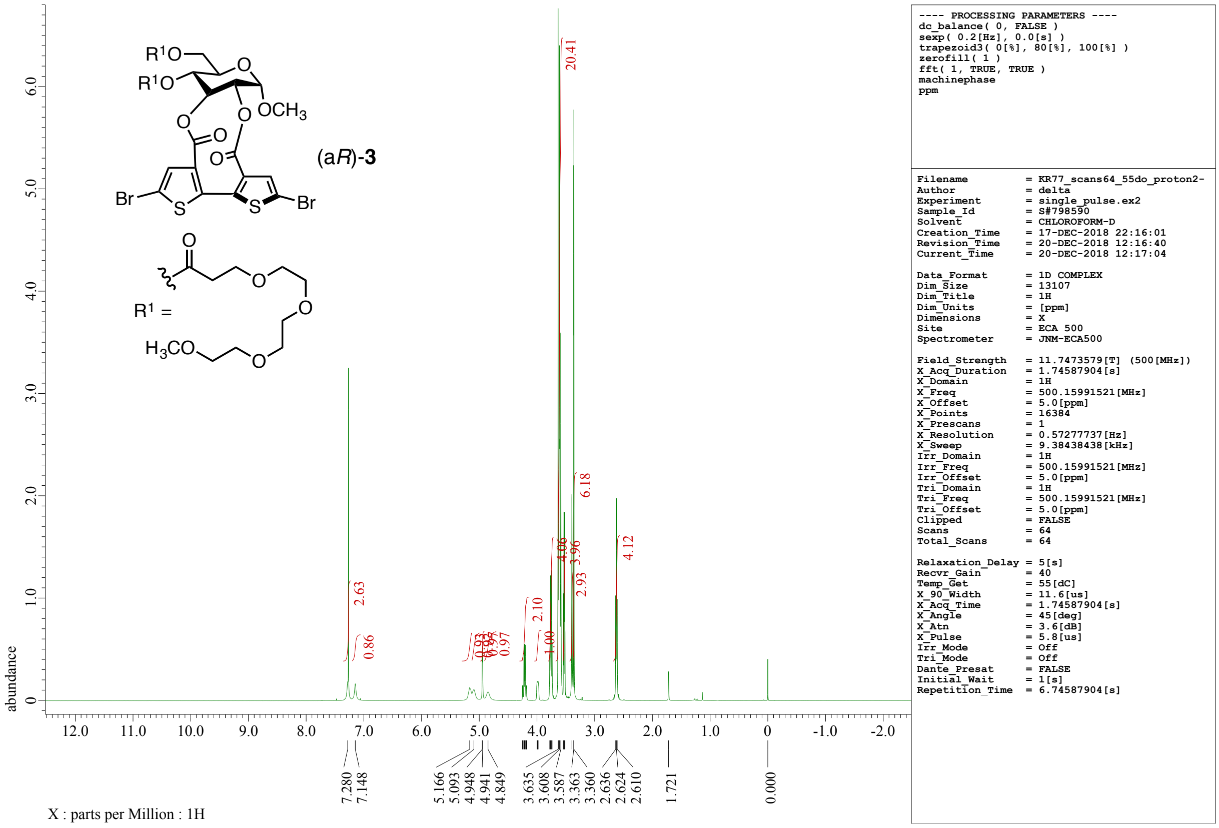


**Fig. S21** $^{1}$H NMR (500 MHz, $CDCl_3$, 55 °C) spectrum of (a*R*)-**3**.

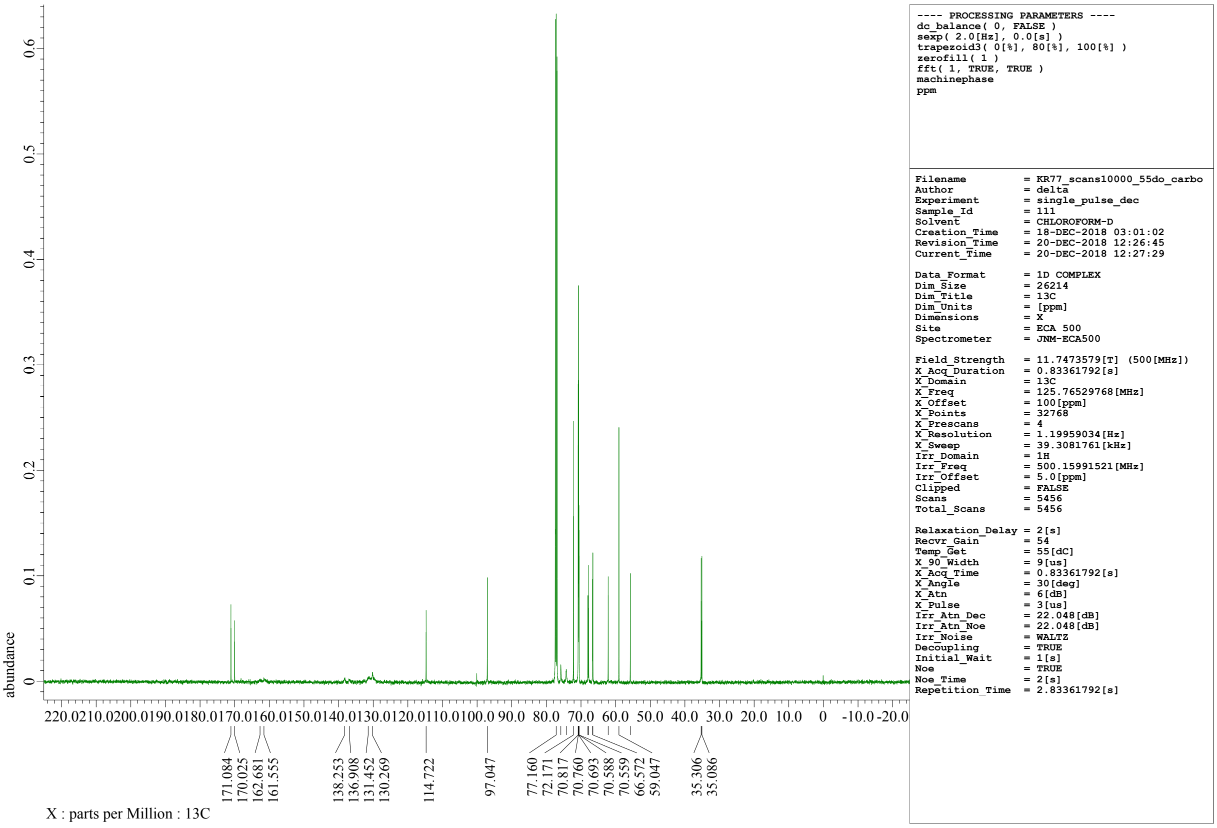


**Fig. S22** $^{13}$C NMR ($CDCl_3$, 126 MHz, 55 °C) spectrum of (a*R*)-**3**.

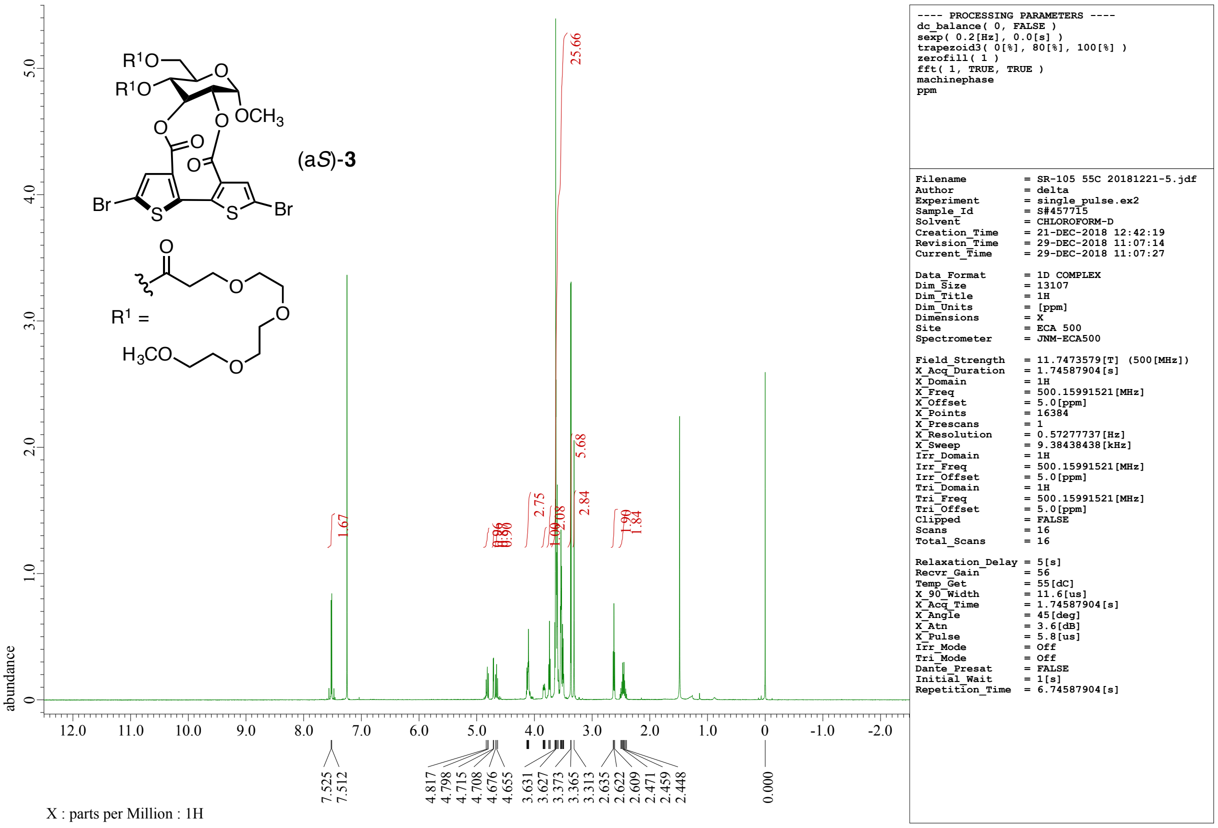


**Fig. S23** $^{1}$H NMR (500 MHz, $CDCl_3$, 55 °C) spectrum of (a*S*)-**3**.

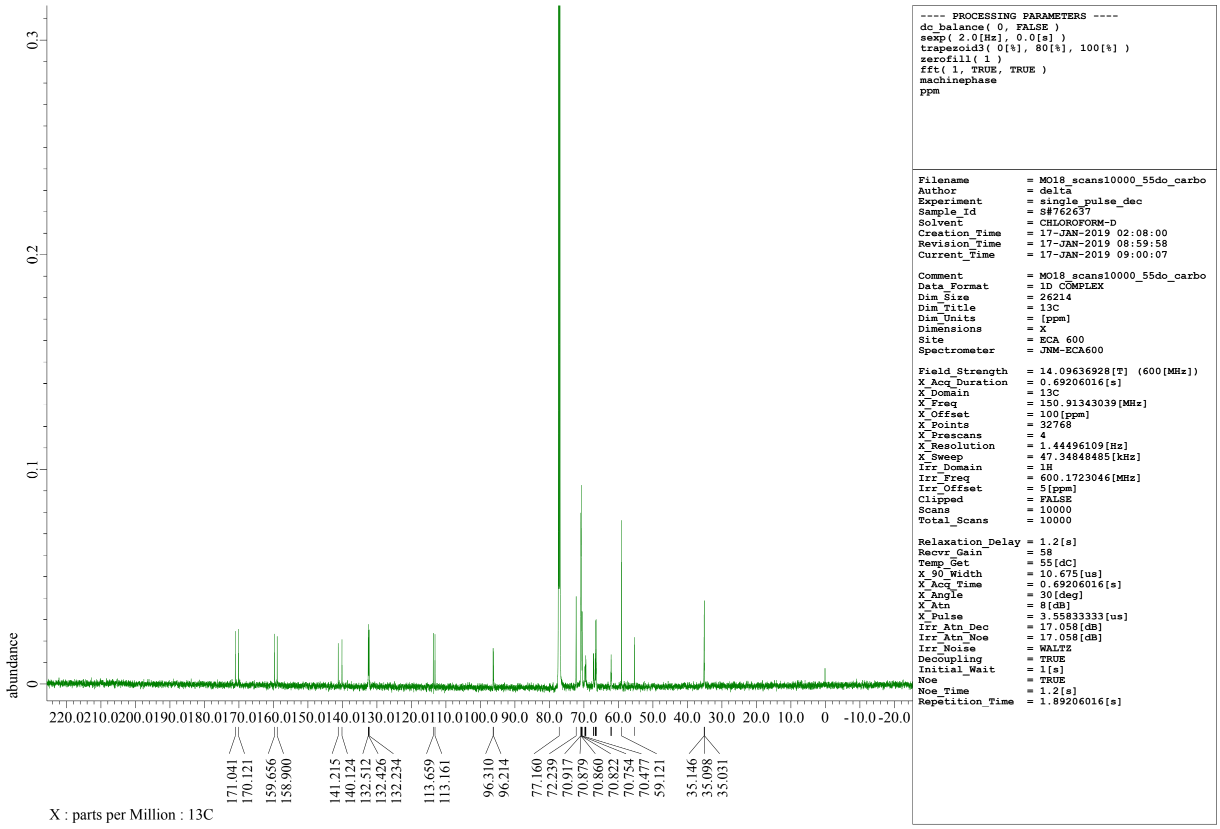


**Fig. S24** $^{13}$C NMR ($CDCl_3$, 151 MHz, 55 °C) spectrum of (a*S*)-**3**.

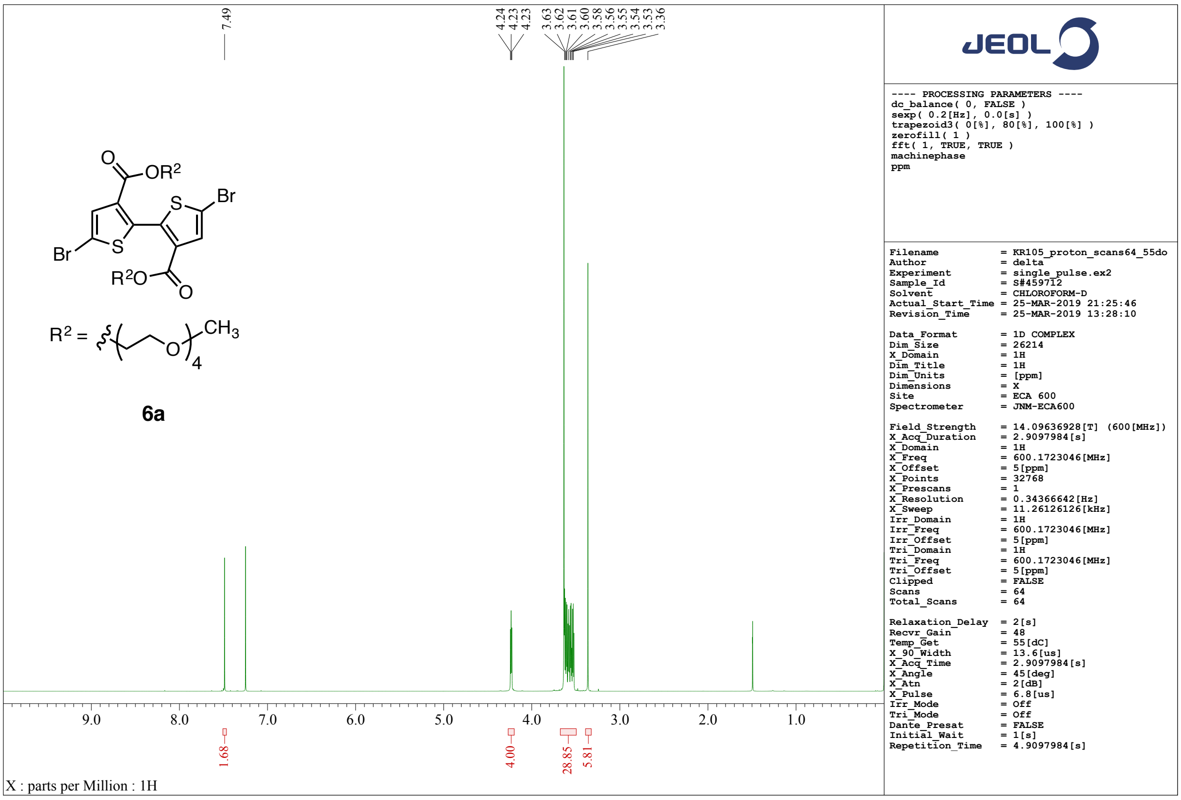


**Fig. S25** $^{1}H$ NMR (600 MHz, $CDCl_3$, 55 °C) spectrum of **6a**.

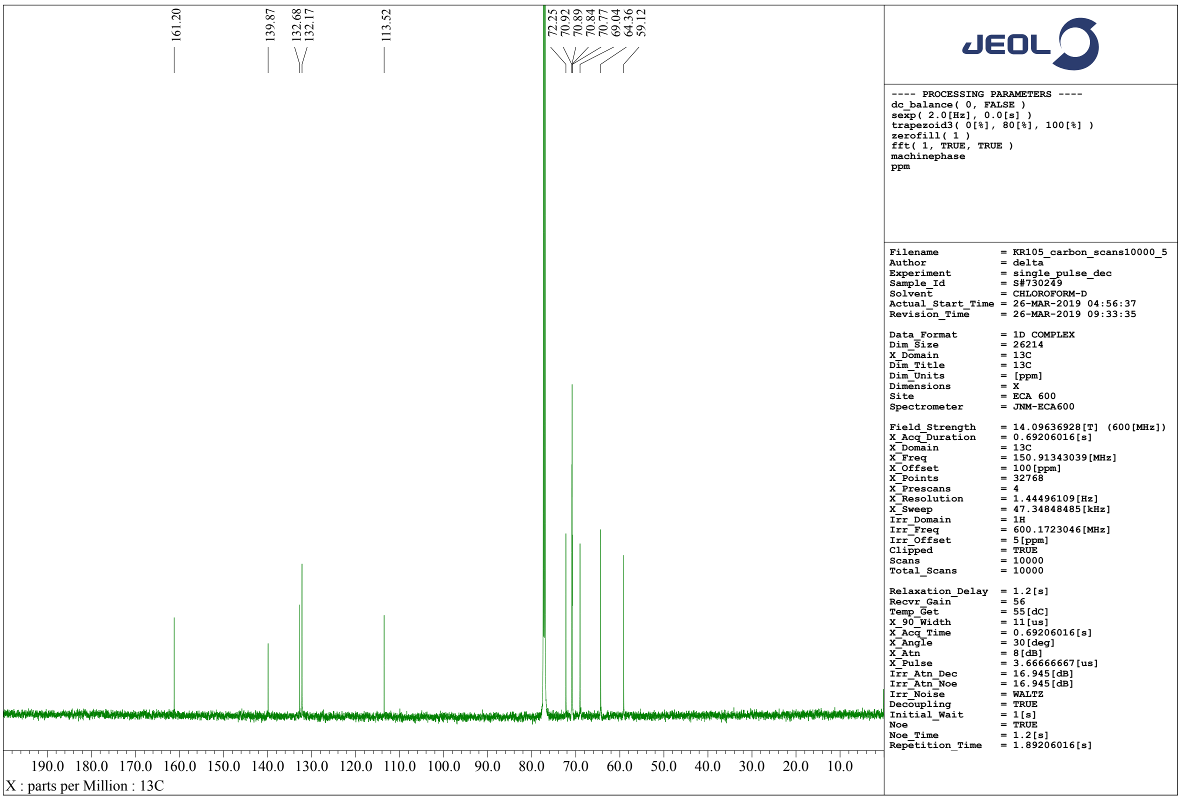


**Fig. S26** $^{13}C$ NMR ($CDCl_3$, 151 MHz, rt) spectrum of **6a**.

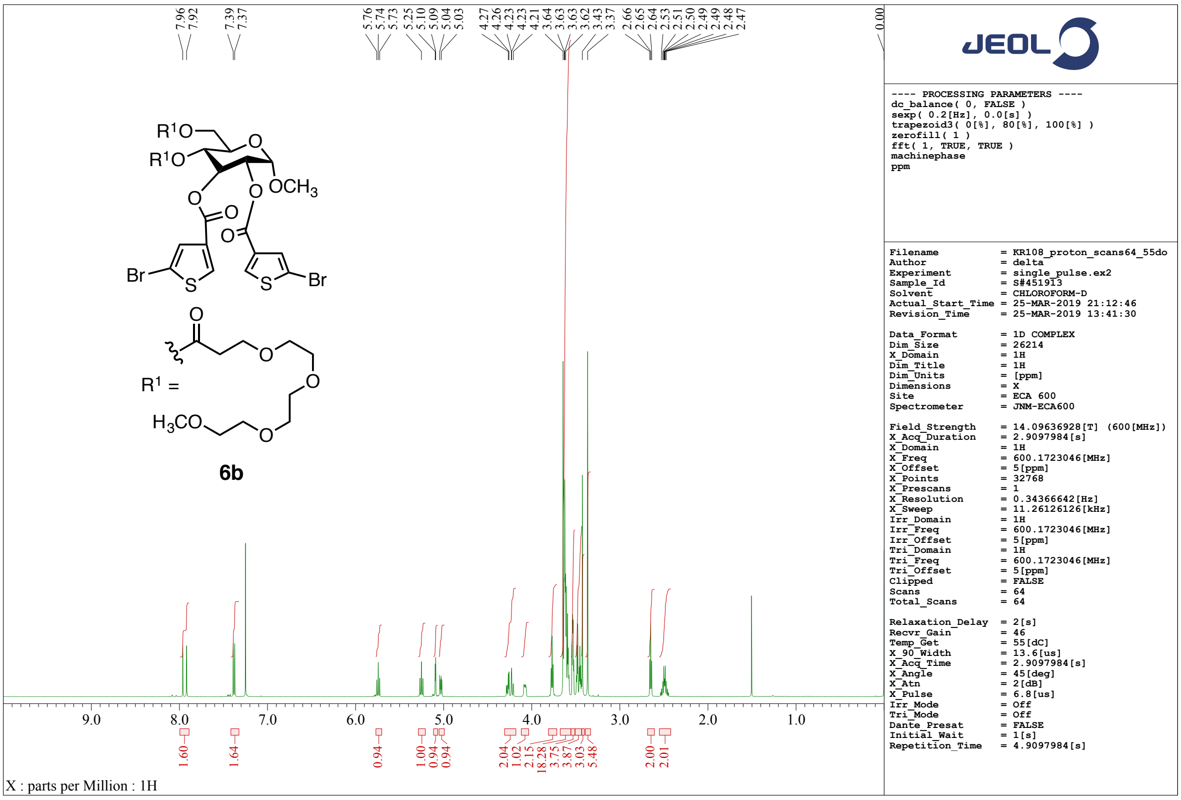

**Fig. S27** $^{1}H$ NMR (600 MHz, $CDCl_3$, 55 °C) spectrum of **6b**.

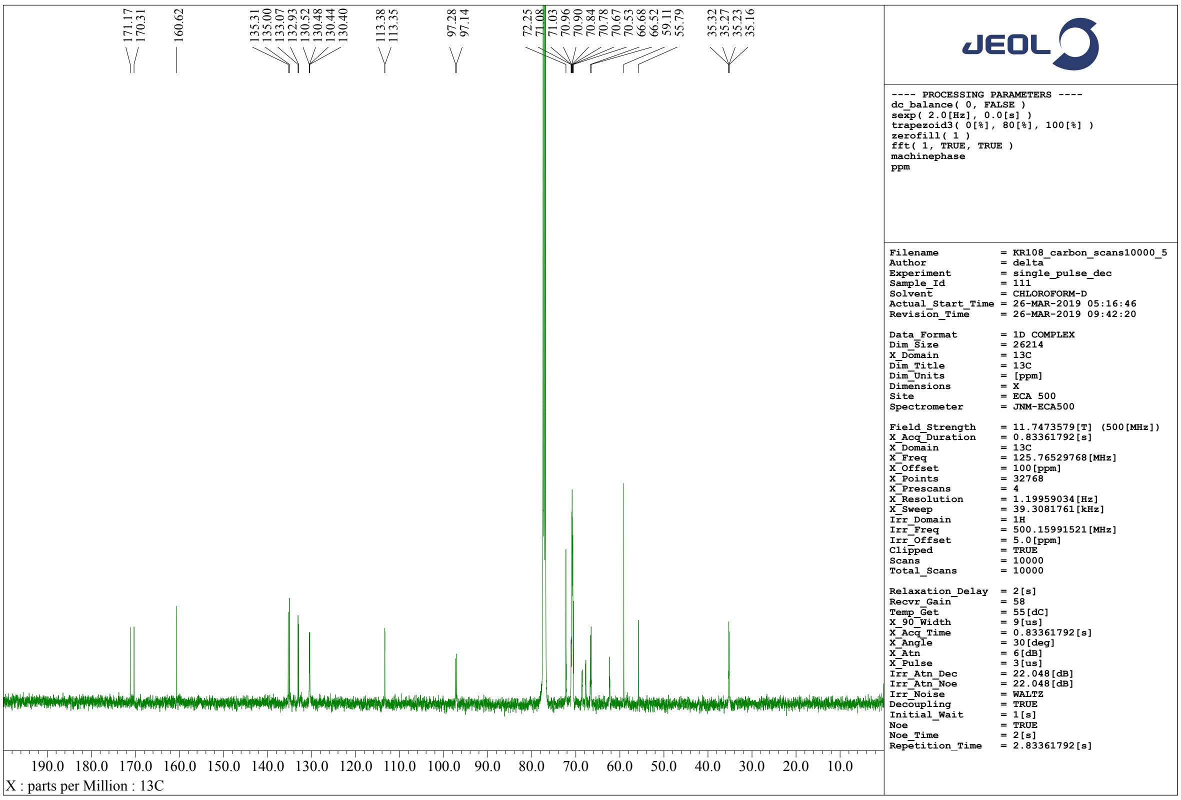

**Fig. S28** $^{13}C$ NMR ($CDCl_3$, 126 MHz, 55 °C) spectrum of **6b**.

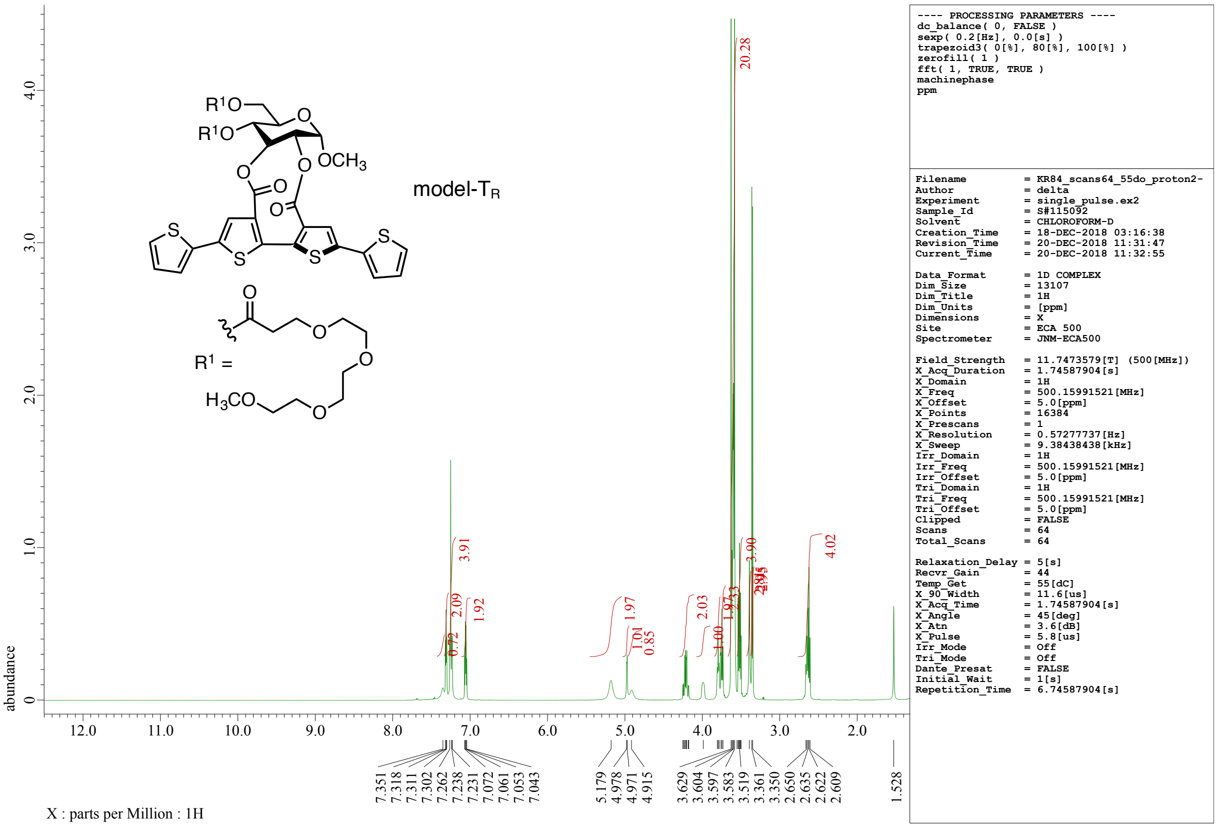


**Fig. S29** $^{1}$H NMR (500 MHz, $CDCl_3$, 55 °C) spectrum of model-T$_R$.

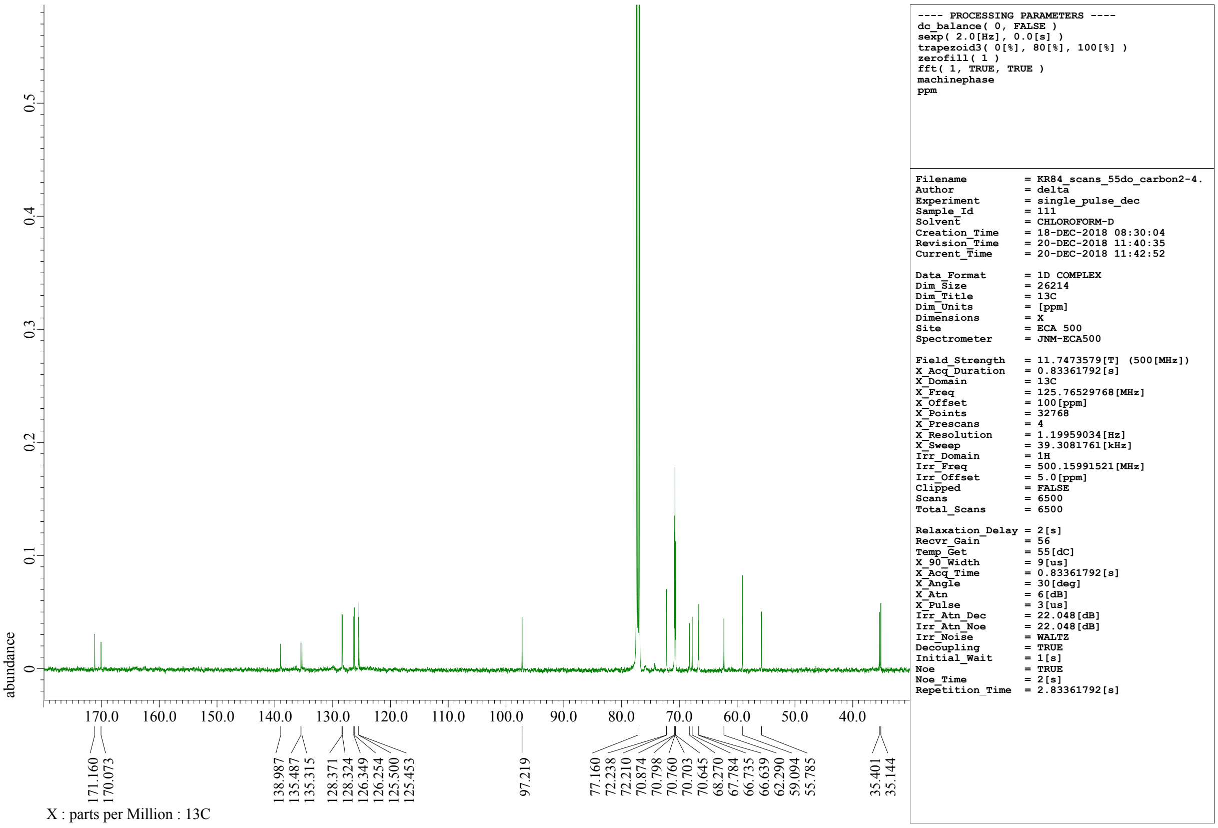


**Fig. S30** $^{13}$C NMR ($CDCl_3$, 126 MHz, 55 °C) spectrum of model-T$_R$.

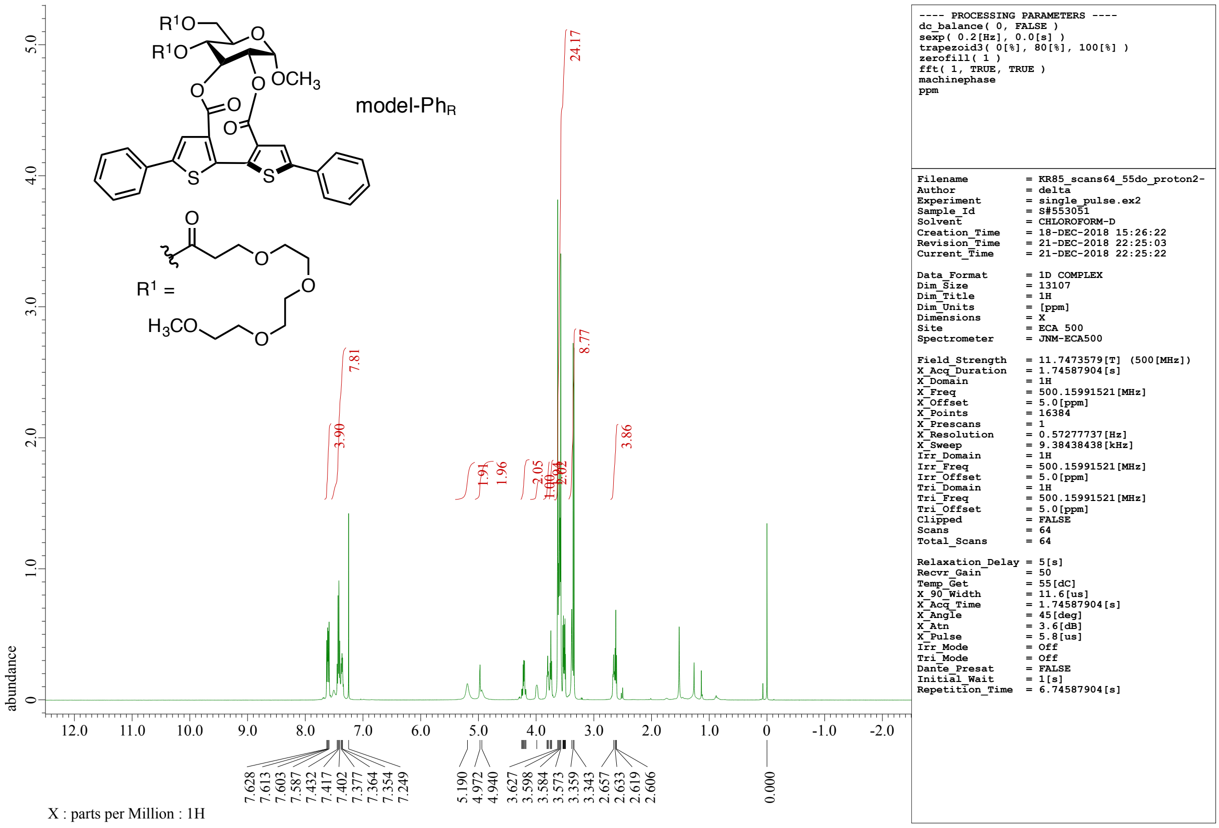


**Fig. S31** $^{1}$H NMR (500 MHz, $CDCl_3$, 55 °C) spectrum of model-Ph$_R$.

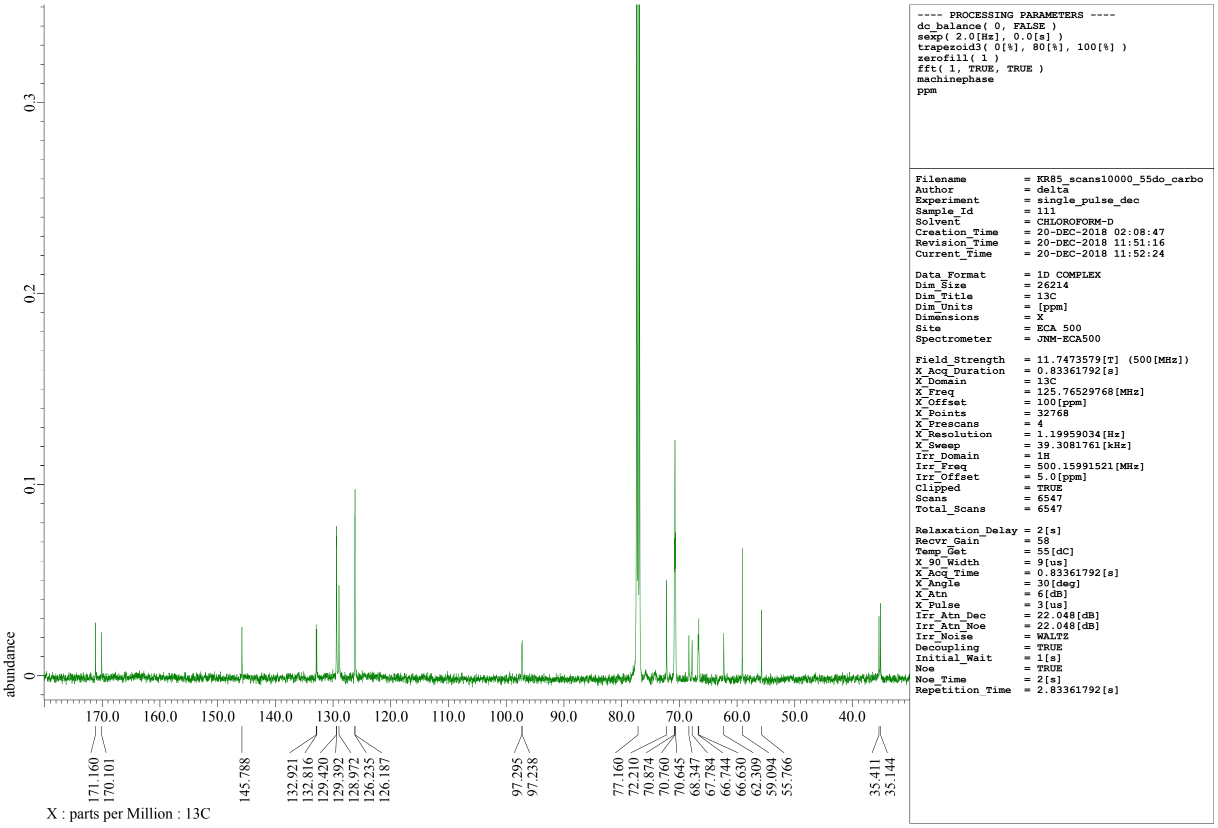


**Fig. S32** $^{13}$C NMR ($CDCl_3$, 126 MHz, 55 °C) spectrum of model-Ph$_R$.

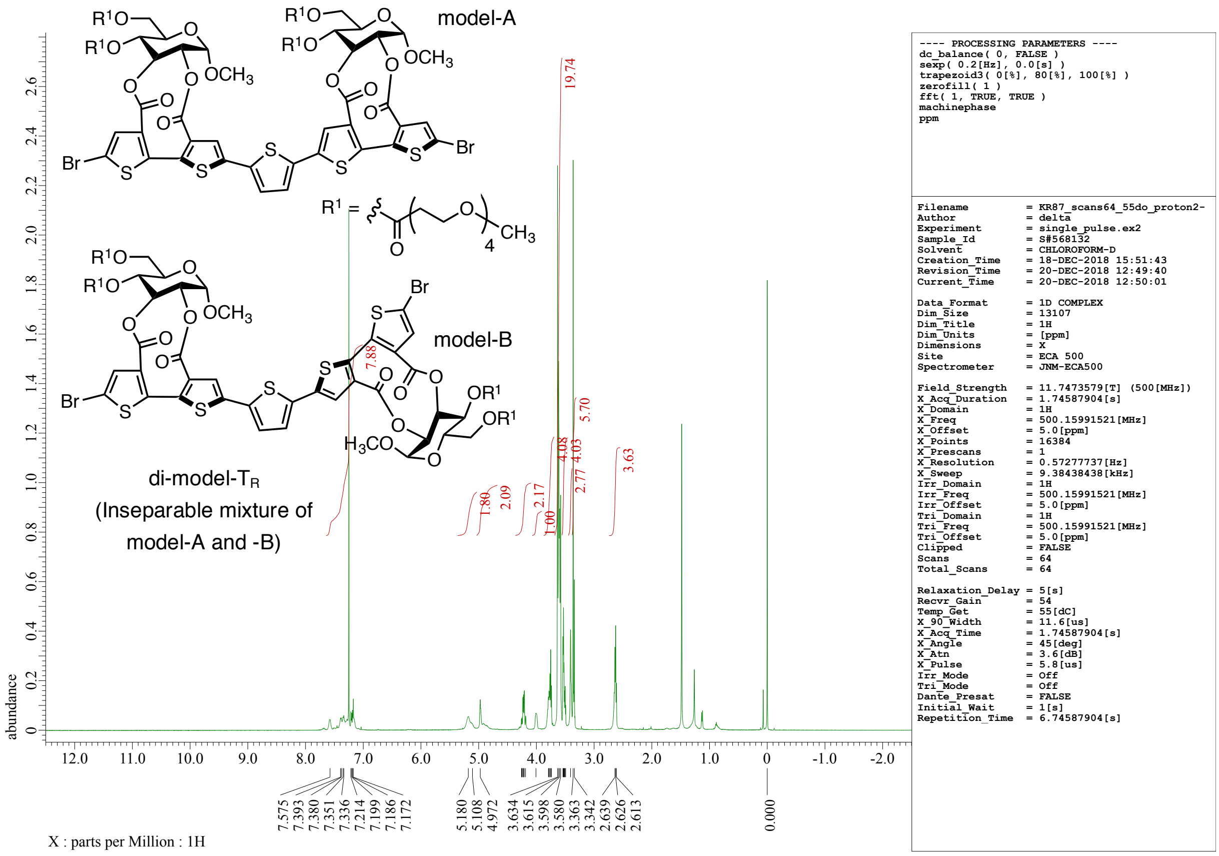


**Fig. S33** $^1$H NMR (500 MHz, CDCl$_3$, 55 °C) spectrum of di-model-T$_R$.

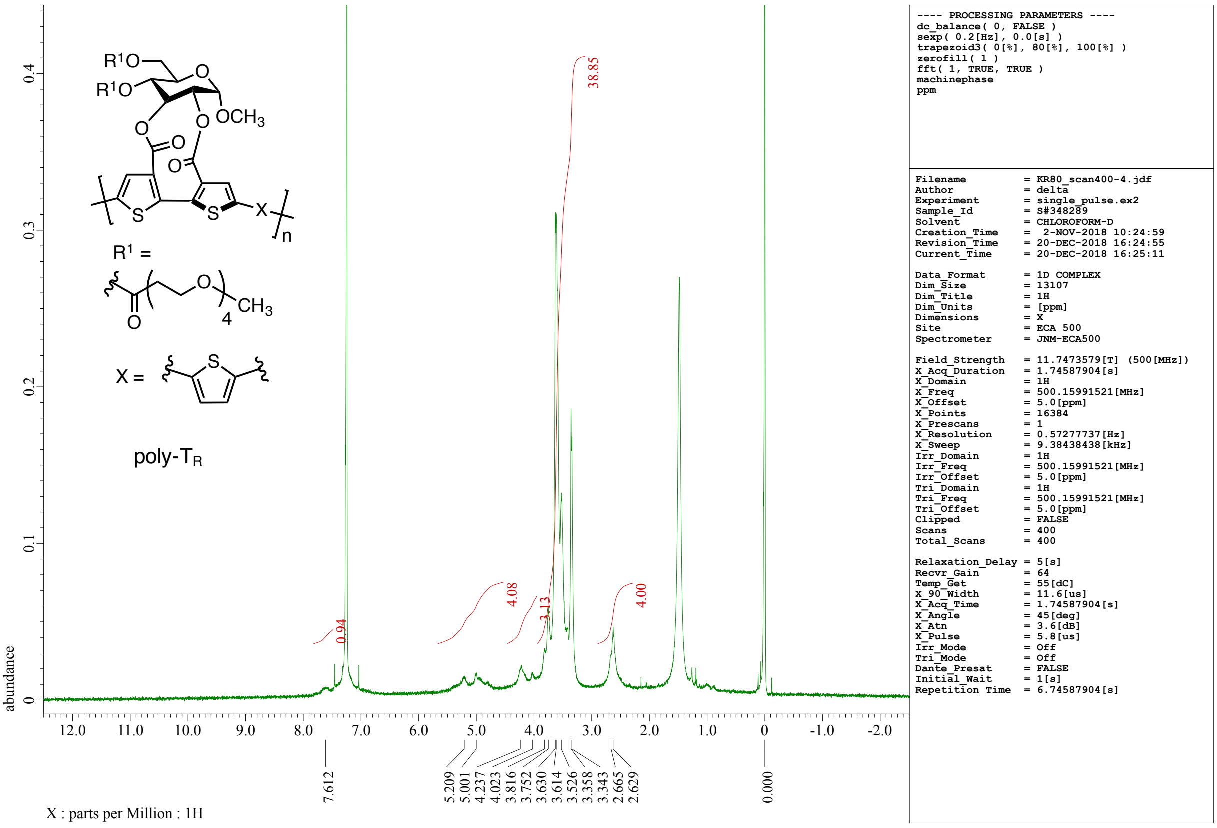


**Fig. S34** $^1$H NMR (500 MHz, CDCl$_3$, 55 °C) spectrum of poly-T$_R$.

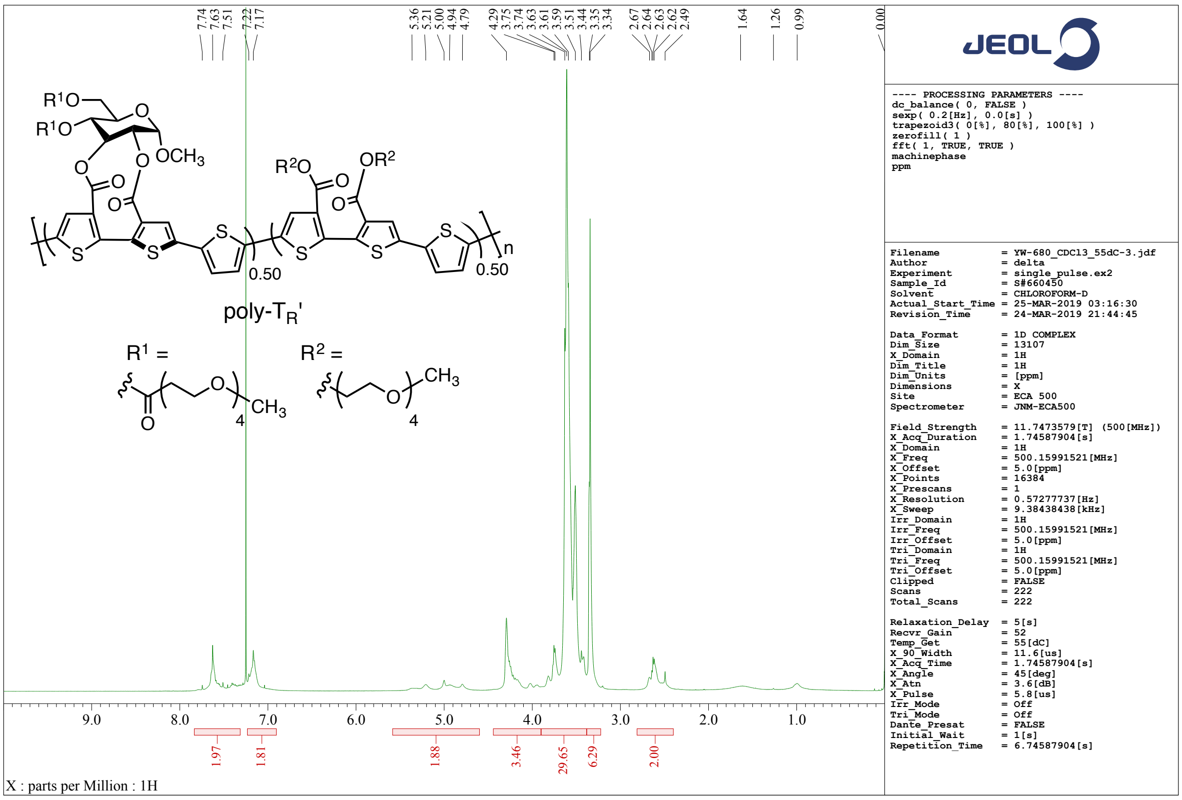

**Fig. S35** $^1$H NMR (500 MHz, $CDCl_3$, 55 °C) spectrum of poly-$T_R$′.

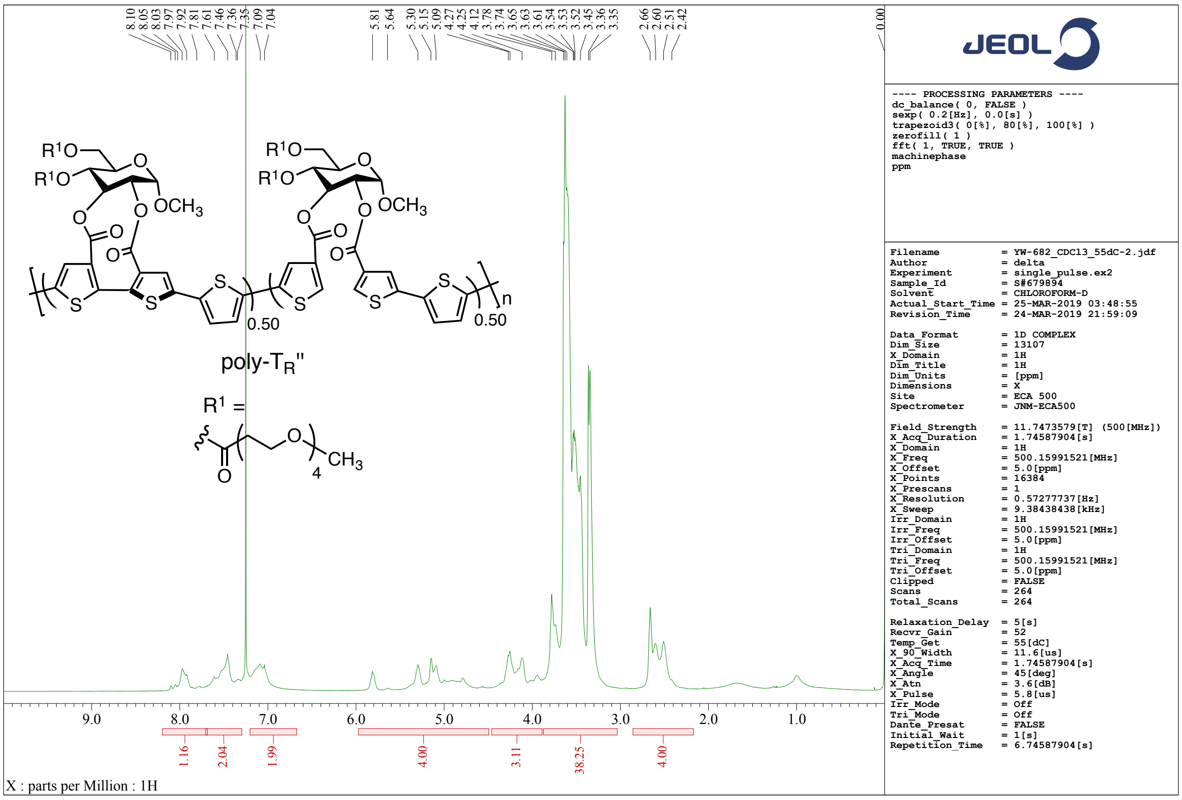

**Fig. S36** $^1$H NMR (500 MHz, $CDCl_3$, 55 °C) spectrum of poly-$T_R$″

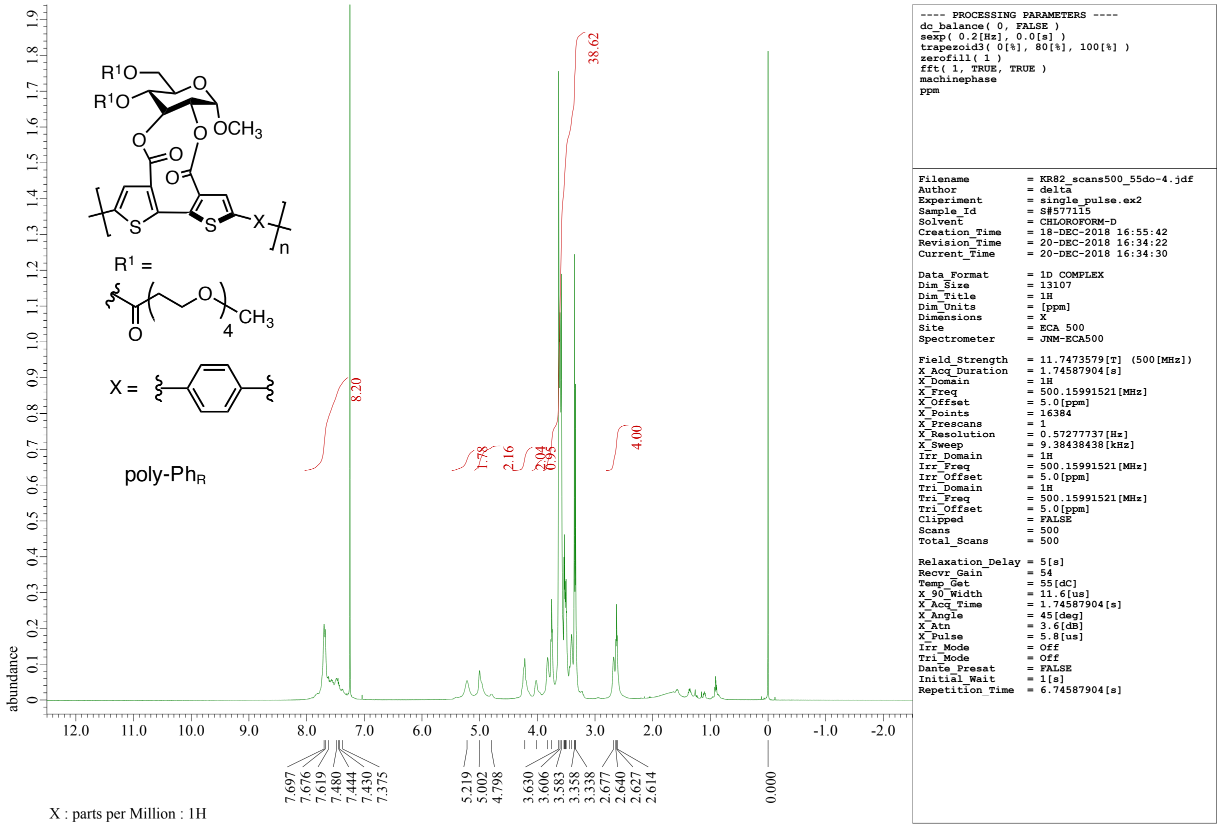


**Fig. S37** $^{1}H$ NMR (500 MHz, $CDCl_3$, 55 °C) spectrum of poly-Ph$_R$.

## Captions for supporting videos

**Video S1.** Animation of all-atom MD simulation in the NVE ensemble (after equilibration at 298 K) of the left-handed helically folded model of poly-$T_R$ (space-filling model for backbone; line model for side chain) in chloroform (line model) at 0–2000 ps as the production run. The poly-$T_R$ backbone is highlighted in purple.

**Video S2.** As Video S1, except that solvent molecules (chloroform) are omitted to simplify the view. The side-view of helical poly-$T_R$ is shown.

**Video S3.** Animation of all-atom MD simulation in the NVE ensemble (after equilibration at 298 K) of the right-handed helically folded model of poly-$T_R$ (space-filling model for backbone; line model for side chain) in chloroform (line model) at 0–2000 ps as the production run. The poly-$T_R$ backbone is highlighted in purple.

**Video S4.** As Video S3, except that solvent molecules (chloroform) are omitted to simplify the view. The side-view of helical poly-$T_R$ is shown.

## References

S1. A. K. Misra, P. Tiwari and S. K. Madhusudan, *Carbohydr. Res.*, 2005, **340**, 325-329.

S2. T. Ikai, S. Minami, S. Awata, S. Shimizu, T. Yoshida, M. Okubo and K. Shinohara, *Polym. Chem.*, 2018, **9**, 5504-5510.
